# Supplementary material for: PTEN Regulates Glucose Transporter Recycling by Impairing SNX27 Retromer Assembly
Source: Cell Rep. 2017 Nov 7;21(6):1655–66. doi: 10.1016/j.celrep.2017.10.053 (PMC5695913; doi:10.1016/j.celrep.2017.10.053)
Supplement: Document S1. Figures S1–S7 [file mmc1.pdf]

Cell Reports, Volume *21*

Supplemental Information

PTEN Regulates Glucose Transporter Recycling by Impairing SNX27 Retromer Assembly

Swapnil Rohidas Shinde and Subbareddy Maddika

Figure S1

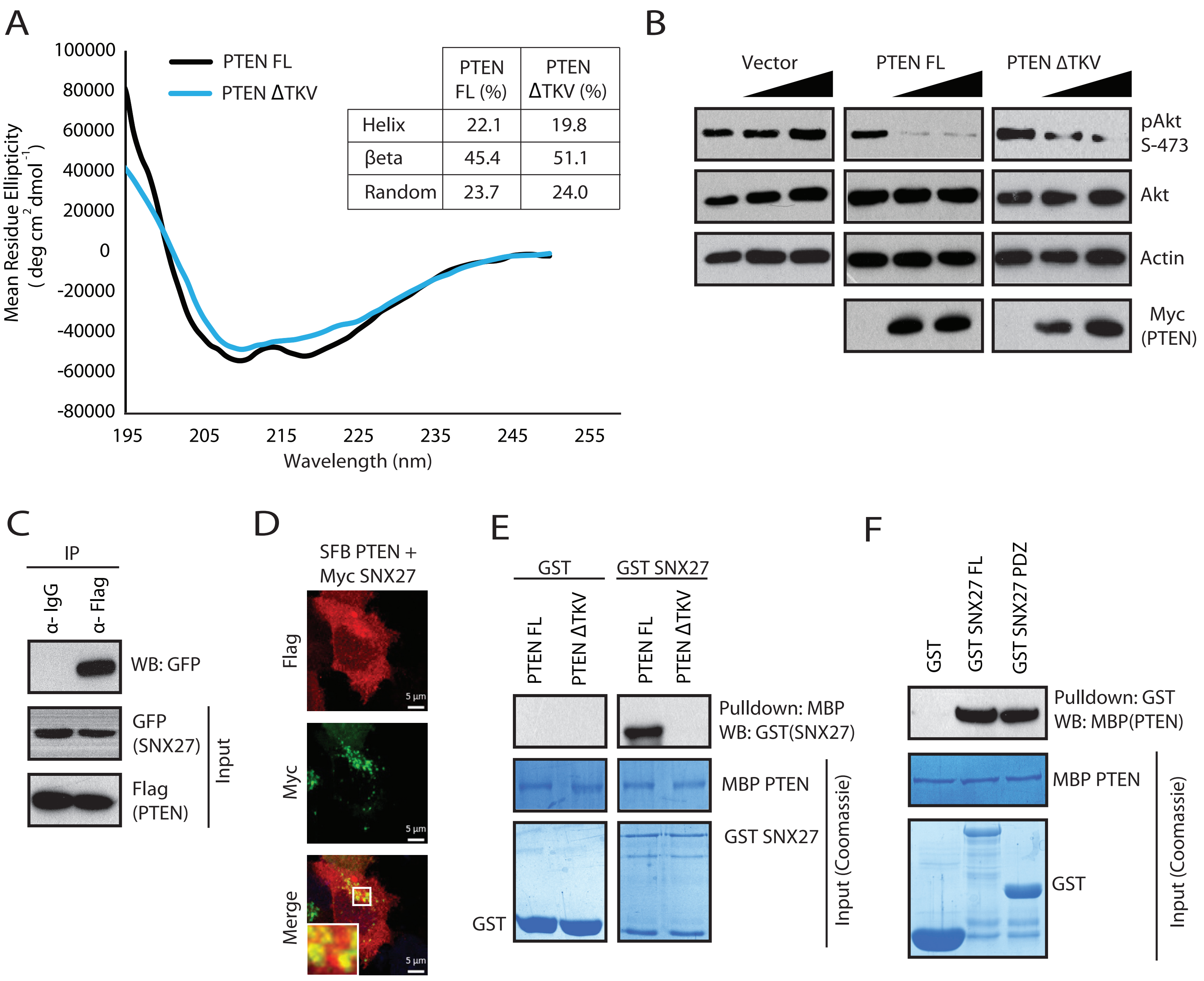

	PTEN FL (%)	PTEN ΔTKV (%)
Helix	22.1	19.8
βeta	45.4	51.1
Random	23.7	24.0

Figure S1, Related to Figure 1.
(A) The CD spectra for full length (FL) PTEN and ΔTKV mutant was shown. **(B)** MDA-MB-231 cells were transfected with vector, PTEN full length (FL) or PTEN ΔTKV mutant. Cell lysates were subjected to immunoblotting analysis and activation of Akt was detected by using a phospho-specific (ser 473) antibody. **(C)** HEK 293T cells were co-transfected with SFB-tagged PTEN along with GFP-SNX27 and the cell lysates were subjected to immunoprecipitation (IP) with either control IgG or Flag antibody and the interaction was determined by western blotting (WB) with GFP antibody. **(D)** Co-localization of exogenously expressed PTEN and SNX27 in cells was shown, scale bar 5 µm. **(E)** Agarose beads immobilized with bacterially expressed recombinant MBP-PTEN or MBP PTEN ΔTKV were incubated with either GST or GST-SNX27 proteins expressed in bacteria. The association of SNX27 with PTEN was detected by immunoblotting with GST antibody. Expression of all recombinant proteins was shown by coomassie staining. **(F)** Glutathione sepharose beads immobilized with bacterially expressed recombinant GST, GST-SNX27 FL or GST-SNX27 ΔPDZ proteins were incubated with bacterially purified recombinant MBP-PTEN. The association of SNX27 with PTEN was detected by immunoblotting with MBP antibody.

Figure S2

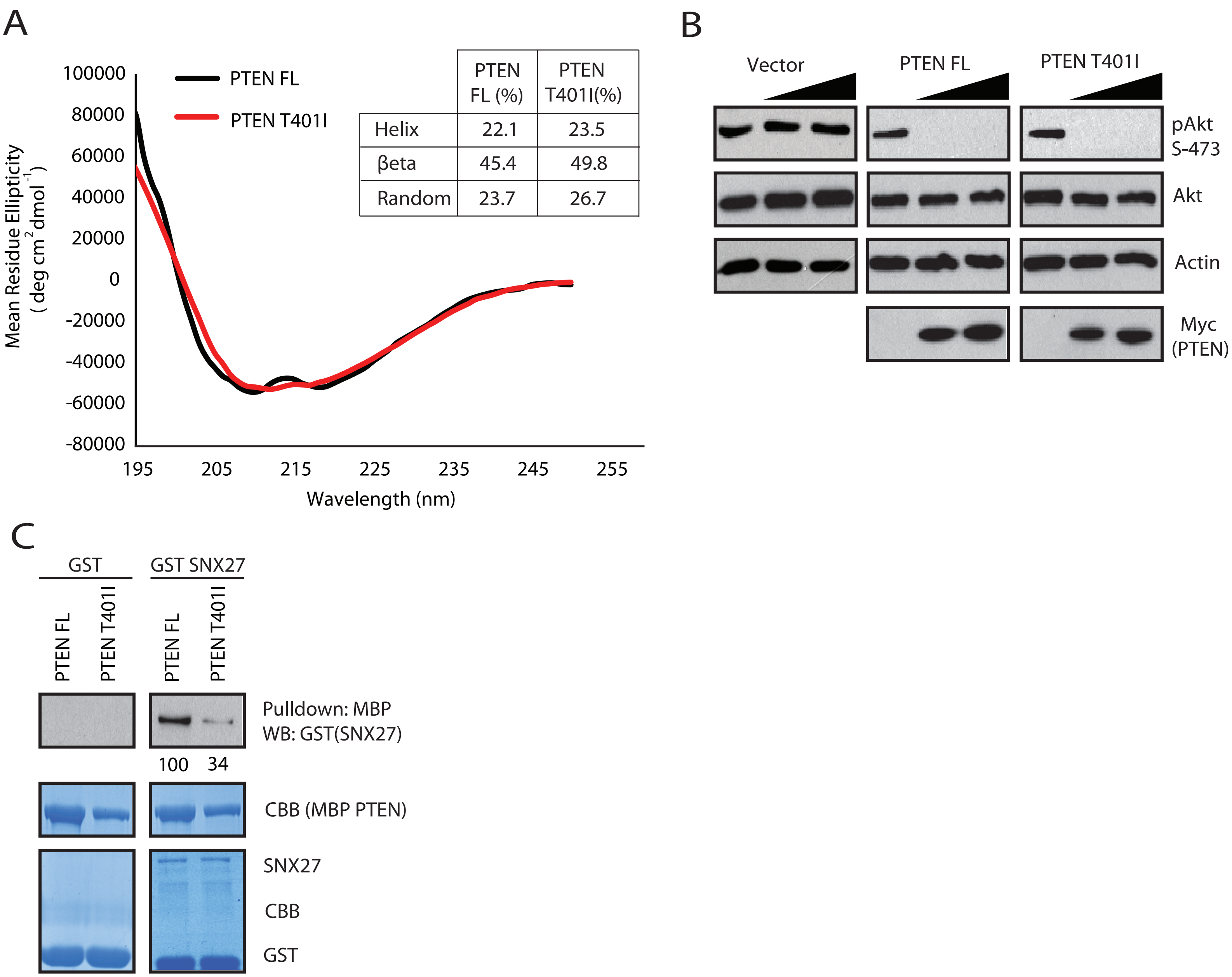

Figure S2, Related to Figure 1.
(A) The CD spectra for full length (FL) PTEN and T401I mutant was shown. **(B)** MDA-MB-231 cells were transfected with vector, PTEN full length (FL) or PTEN T401I mutants. Cell lysates were subjected to immunoblotting analysis and activation of Akt was detected by using a phospho-specific (ser 473) antibody. **(C)** Agarose beads immobilized with bacterially expressed recombinant MBP-PTEN WT or MBP PTEN T401I were incubated with either GST or GST-SNX27 proteins expressed in bacteria. The association of SNX27 with PTEN was detected by immunoblotting with GST antibody. Expression of the all recombinant proteins was shown by coomassie staining.

Figure S3

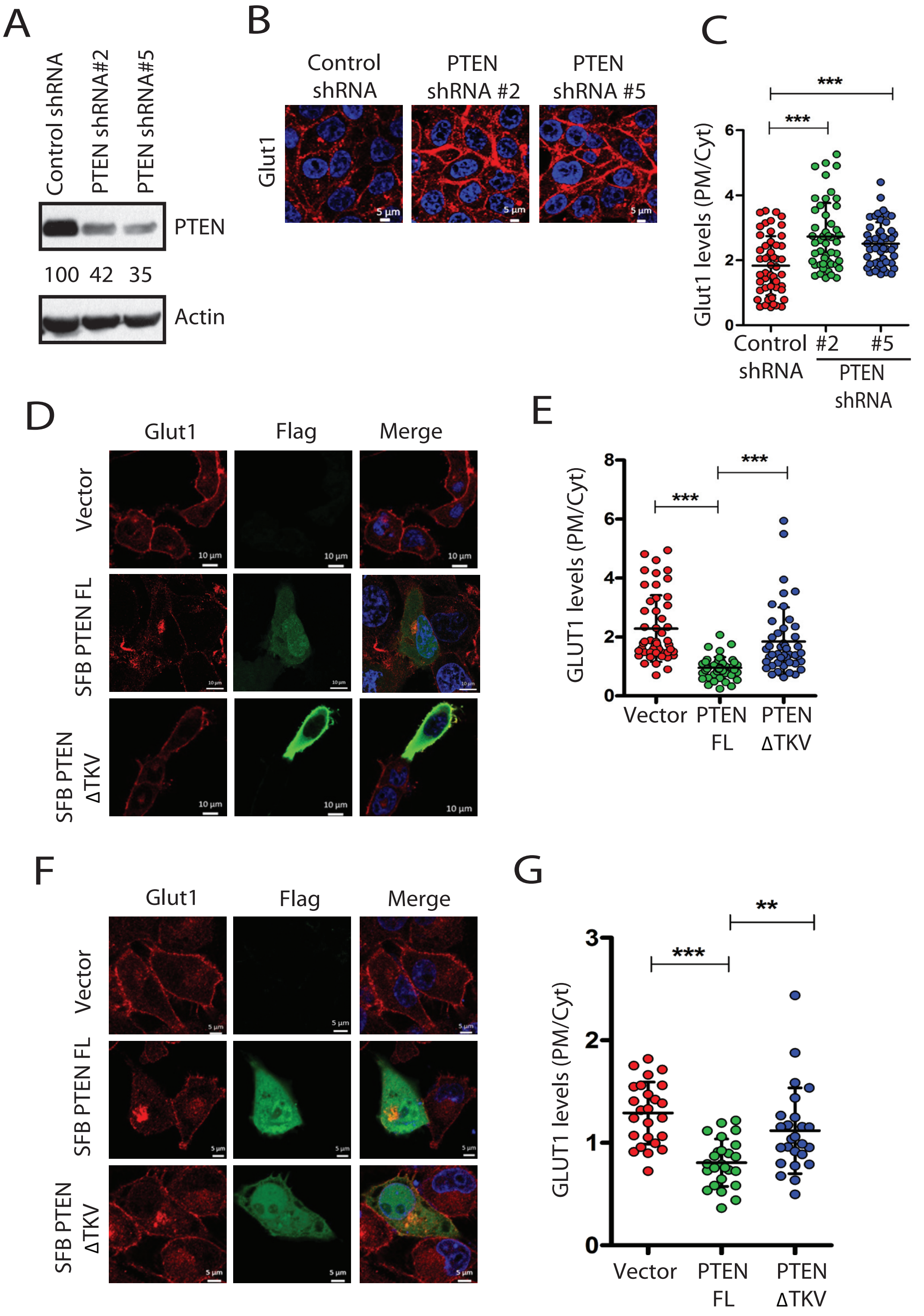

Figure S3, Related to Figure 2.
(A) A representative western blot showing the shRNA mediated knockdown of PTEN in HepG2 cells. **(B)** PTEN depleted HepG2 cells were fixed and imaged using confocal microscope after staining with antibodies against GLUT1. The representative images are shown, Scale bar, 5μm. **(C)** Quantification of plasma membrane (PM) to cytoplasm (Cyt) ratio for GLUT1 intensity per each cell was plotted. Error bars indicate SD (n =50 cells), *** p value (0.001), by Student's t-test. **(D)** Full length (FL) SFB-PTEN or ΔTKV mutant was transfected into MDA-MB231 cells and their effect on GLUT1 membrane levels was determined by confocal imaging after co-staining with Flag and GLUT1 antibodies. The representative images were shown, scale bar, 10 μm. **(E)** Quantification of plasma membrane (PM) to cytoplasm (Cyt) ratio for GLUT1 intensity per each cell was plotted. Error bars indicate SD (n =50 cells), *** p value (0.001), by Student's t-test. **(F)** Full length (FL) SFB PTEN or ΔTKV mutant was transfected into MiaPaCa 2 cells and their effect on GLUT1 membrane levels was determined by confocal imaging after co-staining with Flag and GLUT1 antibodies. The representative images were shown, scale bar, 5 μm. **(G)** Quantification of plasma membrane (PM) to cytoplasm (Cyt) ratio for GLUT1 intensity per each cell was plotted. Error bars indicate SD (n =25 cells), p value (***0.001 or ** 0.01), by Student's t-test.

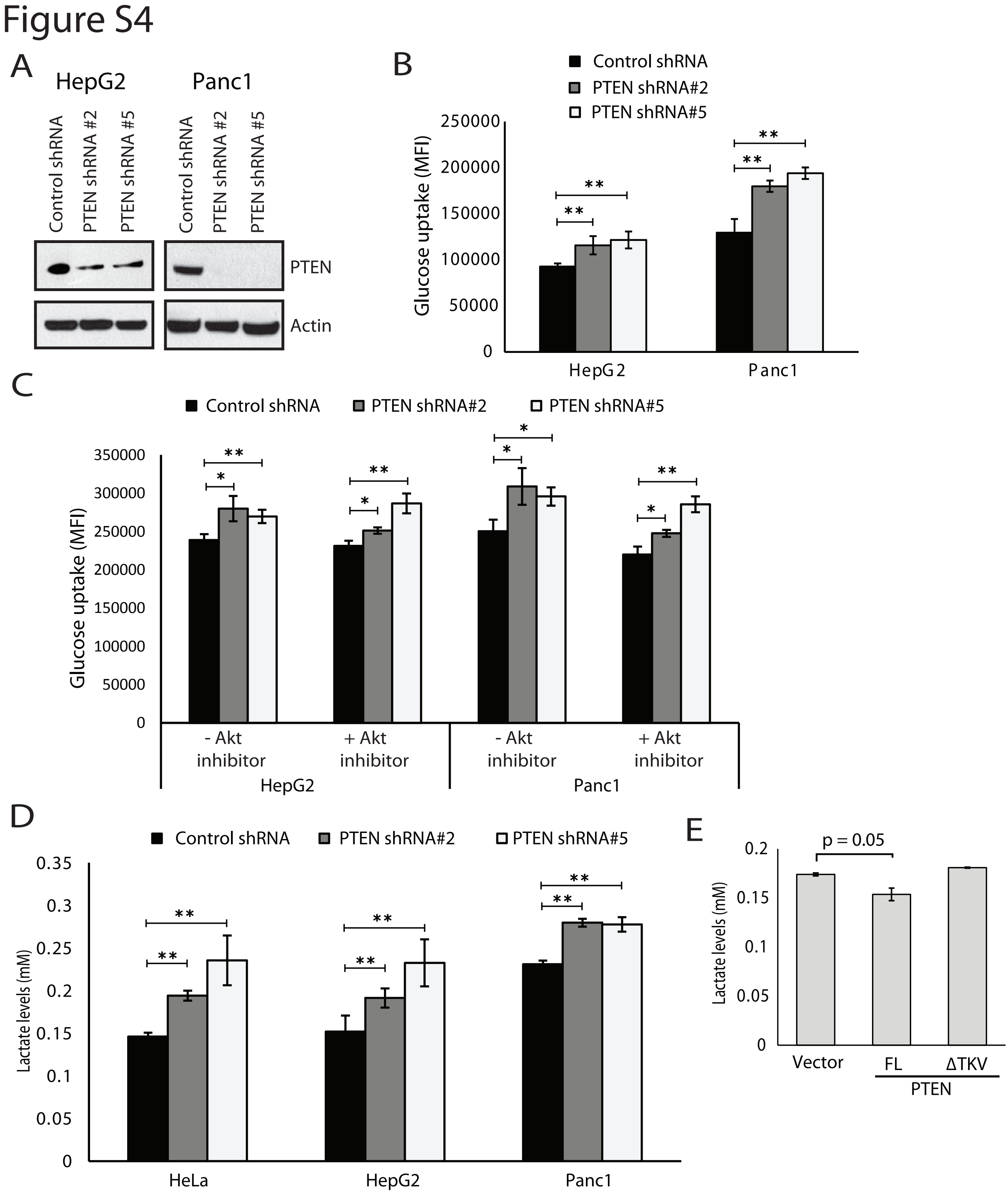

Figure S4, Related to Figure 4.
(A) HepG2 and Panc1 cells transduced with either control or PTEN shRNAs **(B)** were incubated with glucose fluorescent analogue 2-NBDG (100 µg/ml). Glucose uptake in these treated cells was measured. Error bars indicate SD, p value (** 0.01) by Student's t-test. **(C)** HepG2 and Panc1 cells transduced with either control or PTEN shRNAs were treated with Akt inhibitor (MK-2206, 10µM) and glucose uptake in these treated cells was measured. Error bars indicate SD. p value (** 0.01 or * 0.5) by Student's t-test. **(D)** PTEN shRNA transduced cells were lysed and the intracellular lactate concentration was estimated using colorimetry-based assay. Data is expressed as mM concentration of Lactate for three independent experiments. Error bars indicate SD, p value (** 0.01) by Student's t-test. **(E)** MiaPaca2 cells were transfected with SFB- tagged full length (FL) PTEN or PTEN ΔTKV. 24 hours post transfection cells were lysed and intracellular lactate concentration was measured. Data is expressed as mM concentration of Lactate for three independent experiments. Error bars indicate SD, p value (* 0.05) by Student's t-test.

Figure S5

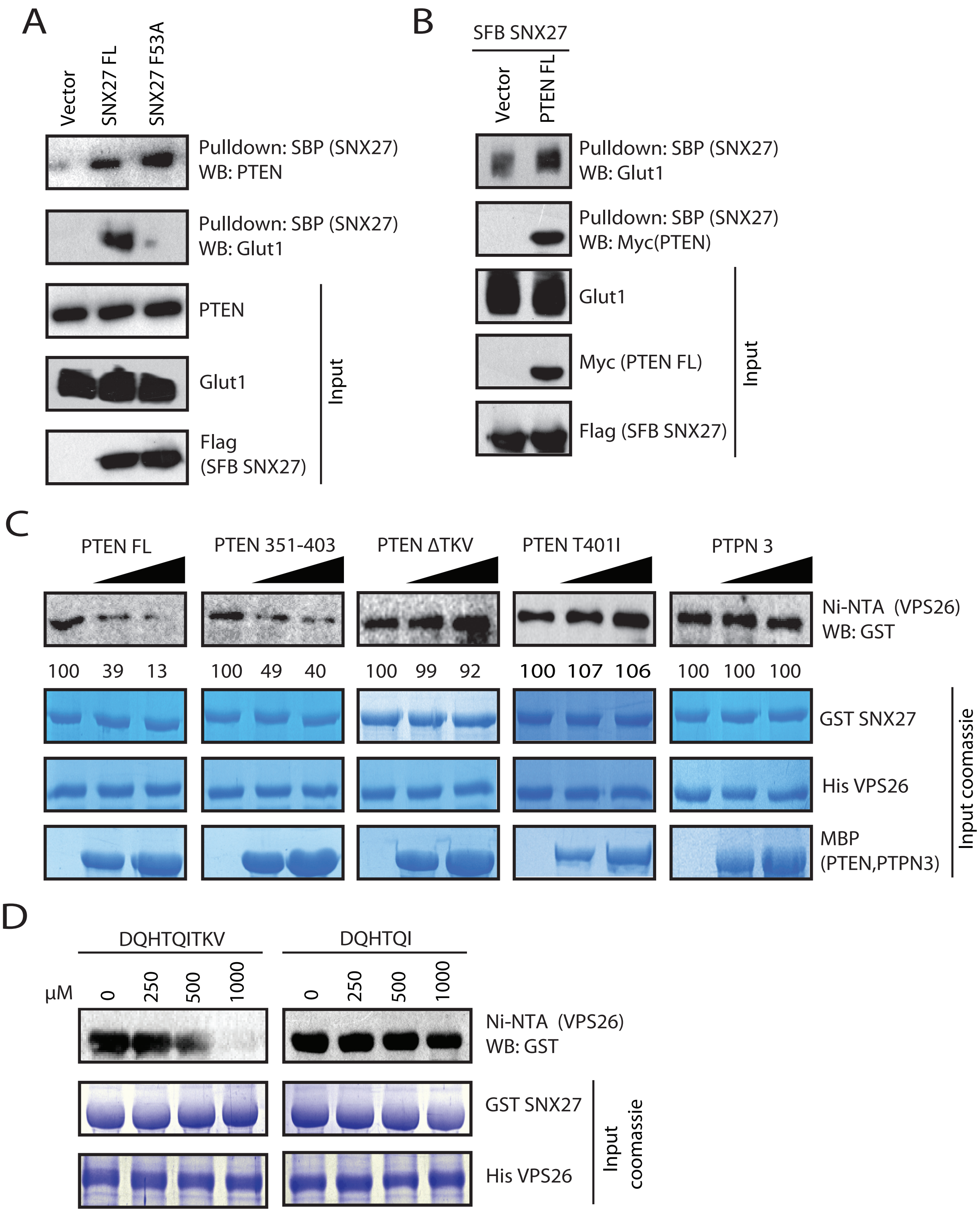

Figure S5, Related to Figure 5.
(A) HEK 293T cells were transfected with the SFB-tagged SNX27 full length (FL) or SNX27 F53A mutant and cell lysates were subjected to pull down with streptavidin beads. The interaction of SNX27 with PTEN and GLUT1 was determined by western blotting with their respective antibodies. **(B)** HEK 293T cells were co-transfected with SFB-tagged SNX27 along with Myc vector or Myc- tagged PTEN and the cell lysates were subjected to pull down with streptavidin beads and interaction of GLUT1 with SNX27 was detected by immunoblotting. **(C)** Bacterially purified recombinant His tagged VPS26 was immobilized on the Ni-NTA beads. Bacterially purified GST-SNX27 was eluted with reduced Glutathione and 5 μM of eluate was incubated with His-tagged VPS26A in presence of increasing PTEN (FL or ΔTKV or T401I or 351-403) or PTPN3. The association of SNX27 with His-VPS26 was determined by western blotting with GST antibody. Expression of all recombinant proteins was shown by coomassie staining. **(D)** The association of recombinant SNX27 with VPS26 in the presence of PTEN derived synthetic peptides was determined by western blotting with GST antibody. Expression of all recombinant proteins was shown by coomassie staining.

Figure S6

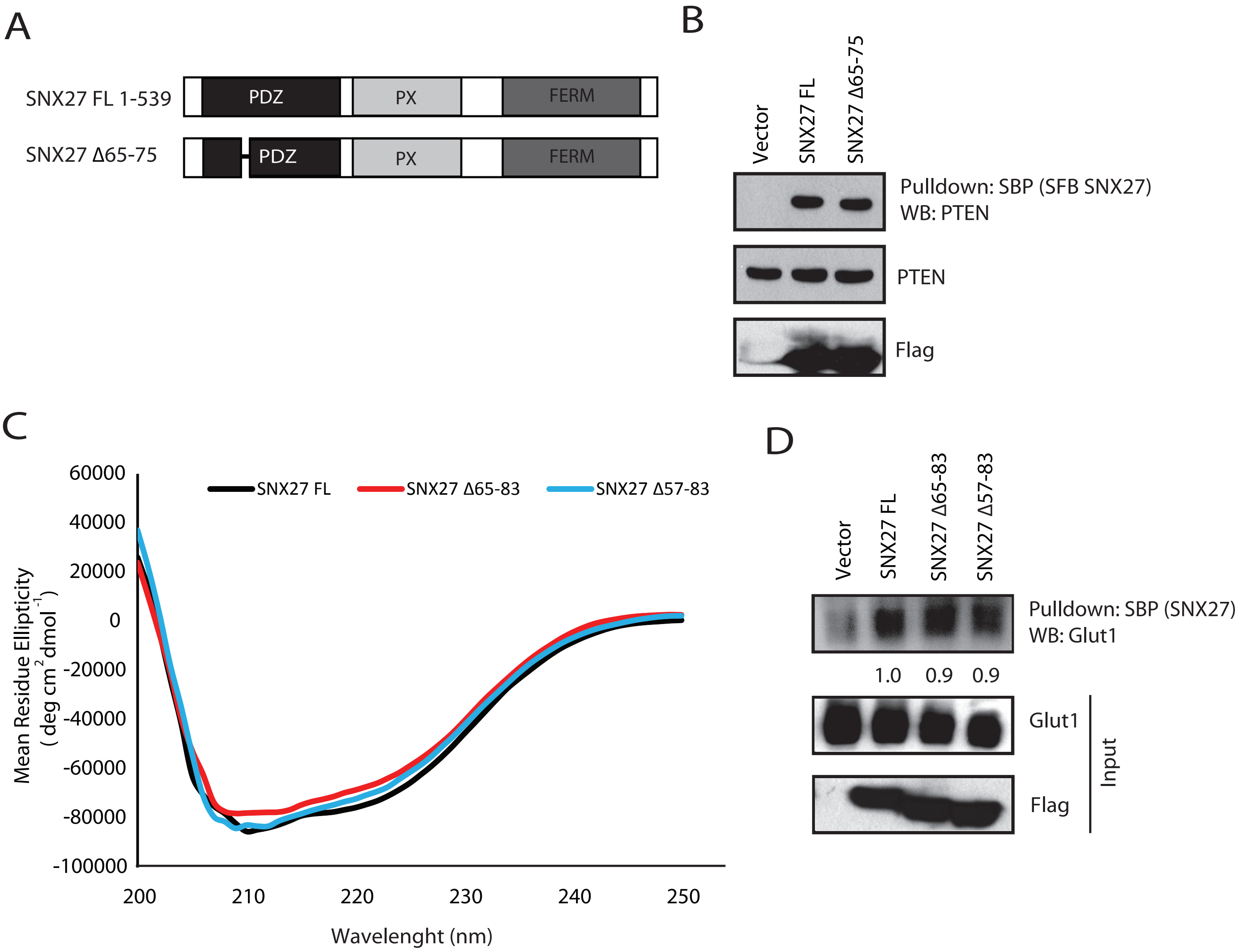

Figure S6, Related to Figure 6.
(A) Schematic representation of N-terminal SFB-tagged SNX27 full length (FL) and SNX27 Δ65-75 mutant. **(B)** Indicated constructs were expressed in HEK 293T cells and the interaction of PTEN with SNX27 was detected by immunoblotting with endogenous PTEN antibody after the cell lysates were pull down with streptavidin beads. The inputs were detected with respective antibodies. **(C)** The CD spectra for full length (FL) SNX27 and the mutants was shown. **(D)** Cells were transfected with the SFB-tagged SNX27 full length (FL) or the indicated mutants and cell lysates were subjected to pull down with streptavidin beads. The interaction of SNX27 with GLUT1 was determined by western blotting.

Figure S7

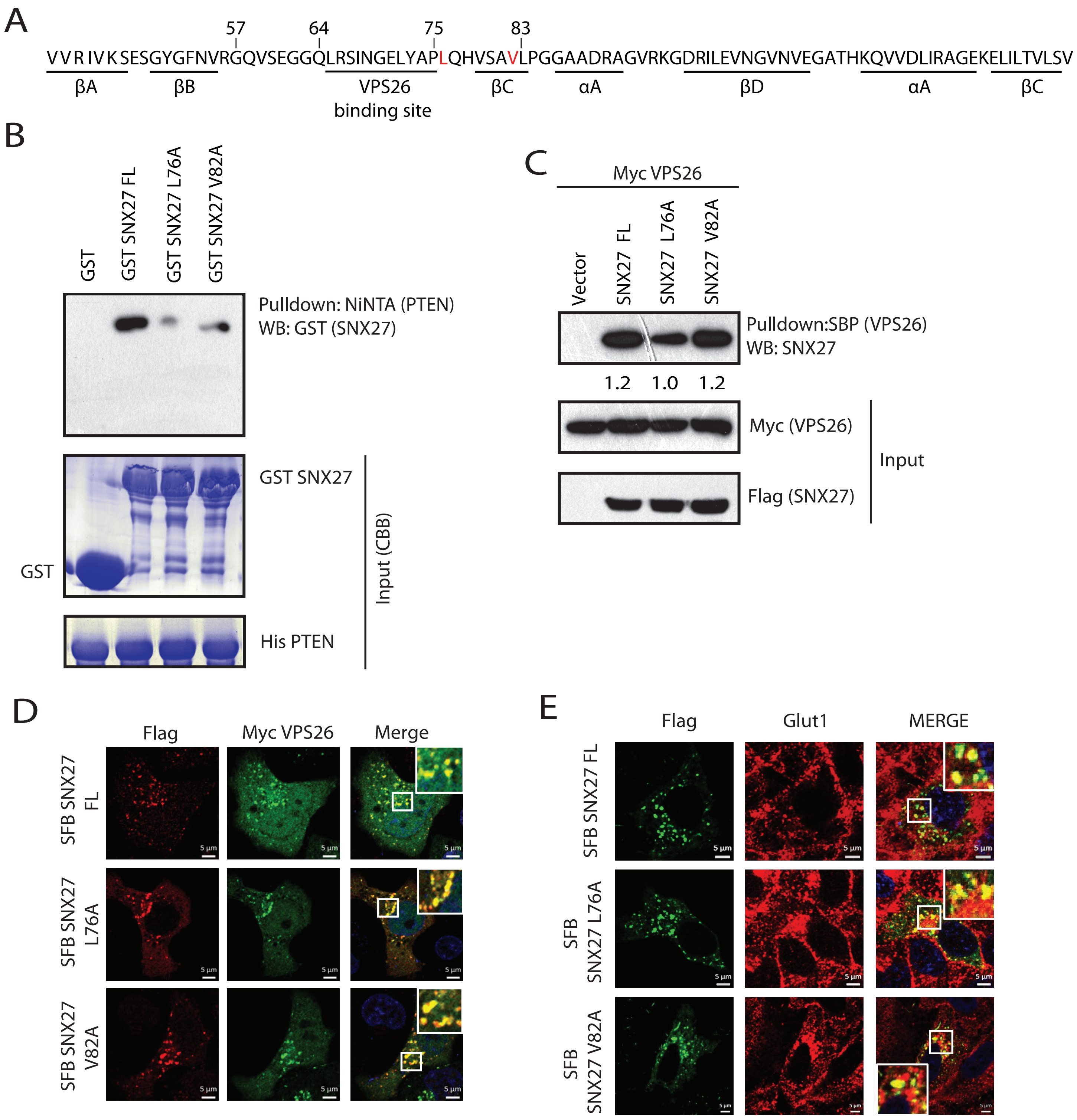

Figure S7, Related to Figure 6.
(A) The sequence of SNX27 PDZ domain is shown along with its VPS26 binding site. The sites of PTEN interaction are highlighted in red (L76 and V82). **(B)** Ni-NTA bound PTEN was incubated with bacterially expressed recombinant GST, GST-SNX27 full length or mutant proteins. The association of SNX27 with PTEN was detected by immunoblotting with GST antibody. Expression of all the recombinant proteins was shown by coomassie staining. **(C)** SFB-tagged SNX27 FL or SNX27 L76A or SNX27 V82A mutants along with Myc VPS26 were transfected in HEK 293T cells. The cell lysates were subjected to pull down with streptavidin beads. The interaction of VPS26 with SNX27 was determined by western blotting Myc antibody. The inputs were blotted with respective antibodies. **(D)** HeLa cells were co-transfected with SFB-SNX27 or mutants along with Myc VPS26. 24 hours post transfection cells were fixed and co-localization of SNX27 and VPS26 was determined by confocal imaging after staining with specific antibodies. **(E)** HeLa cells were co-transfected with SFB-SNX27 or mutants. 24 hours post transfection cells were fixed and co-localization of SNX27 with GLUT1 was determined by confocal imaging after staining with specific antibodies.
