# Supplementary material for: PTEN Regulates Glucose Transporter Recycling by Impairing SNX27 Retromer Assembly
Source: Cell Rep. 2017 Nov 7;21(6):1655–66. doi: 10.1016/j.celrep.2017.10.053 (PMC5695913; doi:10.1016/j.celrep.2017.10.053)
Supplement: Document S2. Article plus Supplemental Information [file mmc2.pdf]

Cell Reports

Article

PTEN Regulates Glucose Transporter Recycling by Impairing SNX27 Retromer Assembly

Graphical Abstract

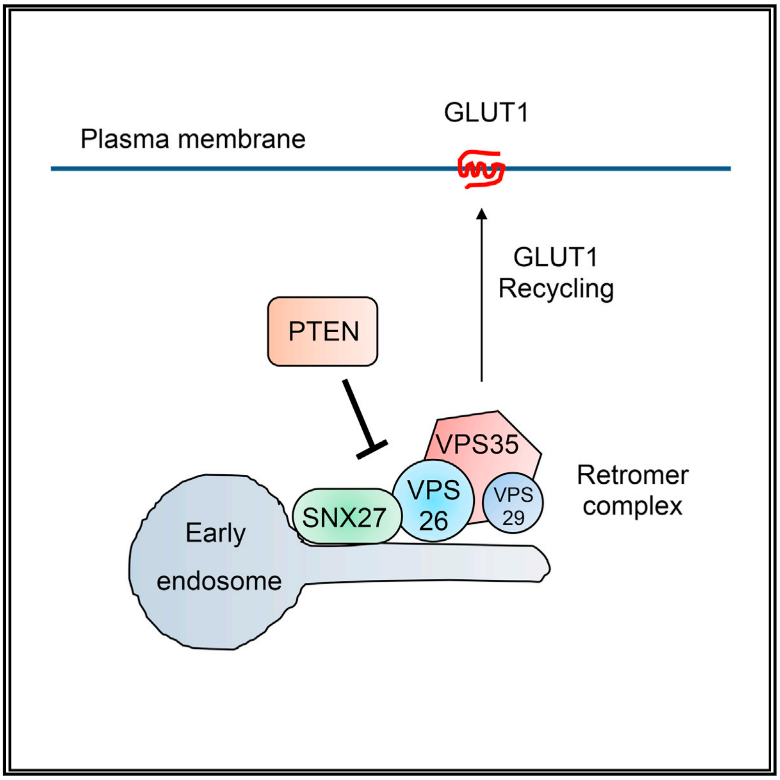

Highlights

- PTEN inhibits the assembly of the SNX27-VPS26 retromer complex
- PTEN hinders retromer assembly in a phosphatase-independent manner
- Endosome-to-plasma membrane recycling of GLUT1 is inhibited by PTEN
- PTEN binding with SNX27 impairs cellular glucose uptake

Authors

Swapnil Rohidas Shinde,
Subbareddy Maddika

Correspondence

msreddy@cdfd.org.in

In Brief

Shinde et al. identify a critical role for the tumor suppressor PTEN in the regulation of glucose uptake by cells. PTEN binds SNX27 and hinders its access to the VPS26 retromer complex, preventing recycling of the glucose transporter GLUT1 to the plasma membrane, which leads to impaired cellular glucose uptake.

Shinde & Maddika, 2017, Cell Reports *21*, 1655–1666
November 7, 2017 © 2017 The Author(s).
https://doi.org/10.1016/j.celrep.2017.10.053

OPEN ACCESS
CellPress

PTEN Regulates Glucose Transporter Recycling by Impairing SNX27 Retromer Assembly

Swapnil Rohidas Shinde[1,2] and Subbareddy Maddika[1,3,*]
[1]Laboratory of Cell Death & Cell Survival, Centre for DNA Fingerprinting and Diagnostics (CDFD), Hyderabad 500001, Telangana, India
[2]Graduate Studies, Manipal University, Manipal 576104, Karnataka, India
[3]Lead Contact
*Correspondence: msreddy@cdfd.org.in
https://doi.org/10.1016/j.celrep.2017.10.053

SUMMARY

The tumor suppressor PTEN executes cellular functions predominantly through its phosphatase activity. Here we identified a phosphatase-independent role for PTEN during vesicular trafficking of the glucose transporter GLUT1. PTEN physically interacts with SNX27, a component of the retromer complex that recycles transmembrane receptors such as GLUT1 from endosomes to the plasma membrane. PTEN binding with SNX27 prevents GLUT1 accumulation at the plasma membrane because of defective recycling and thus reduces cellular glucose uptake. Mechanistically, PTEN blocks the association of SNX27 with VPS26 and thereby hinders assembly of a functional retromer complex during the receptor recycling process. Importantly, we found a PTEN somatic mutation (T401I) that is defective in disrupting the association between SNX27 and VPS26, suggesting a critical role for PTEN in controlling optimal GLUT1 levels at the membrane to prevent tumor progression. Together, our results reveal a fundamental role of PTEN in the regulation of the SNX27 retromer pathway, which governs glucose transport and might contribute to PTEN tumor suppressor function.

INTRODUCTION

PTEN (phosphatase and tensin homolog deleted on chromosome 10), a dual-specificity phosphatase, is encoded by chromosome 10q23, a hotspot for loss of heterozygosity in many advanced-stage human cancers (Ali et al., 1999; Li et al., 1997; Myers et al., 1997). In addition, PTEN germline mutations are associated with several inherited syndromes (such as Cowden syndrome, Bannayan-Riley-Ruvalcaba syndrome, and Proteus syndrome) characterized by hamartomatous growth (Liaw et al., 1997). Also, various somatic mutations of PTEN are observed in a wide cancer spectrum, including breast, prostate, kidney, and brain tumors (Li et al., 1997). Enzymatically, PTEN catalyzes the conversion of phosphatidylinositol-3,4,5-trisphosphate (PIP3) to phosphatidylinositol-4,5-bisphosphate (PIP2) (Maehama and Dixon, 1998; Myers et al., 1997). In response to growth factors, phosphatidylinositol 3-kinase (PI3K) activates Akt signaling by catalyzing the formation of the lipid second messenger PIP3. PTEN dephosphorylates PIP3 and, thereby, controls the activation of the PI3K/AKT pathway (Maehama and Dixon, 1998; Myers et al., 1997; Stambolic et al., 1998). In addition to its lipid phosphatase activity, PTEN dephosphorylates multiple protein substrates, such as Shc, EG5, IRS1, and Rab7, to exert cellular functions, including cell motility, mitosis, insulin signaling, and endosome maturation (Gu et al., 1999; He et al., 2016; Shi et al., 2014; Shinde and Maddika, 2016).

The majority of tumor-associated PTEN mutations are found in its phosphatase domain; however, some mutations occur outside the catalytic domain (Ali et al., 1999; Di Cristofano and Pandolfi, 2000; Duerr et al., 1998), possibly suggesting phosphatase-independent roles of PTEN in tumor suppression. However, studies of PTEN phosphatase-independent cellular functions are limited. In this study, we uncovered an essential phosphatase-independent role of PTEN in regulating glucose transport. We identified SNX27 as a PTEN functional partner in the cell. SNX27 associates with the retromer complex (a heterotrimer of VPS26-VPS29-VPS35) and promote endosome-to-membrane trafficking of plasma membrane receptors (Lauffer et al., 2010; Lunn et al., 2007; Temkin et al., 2011). SNX27 utilizes both canonical and noncanonical PDZ interfaces to simultaneously engage type 1 PDZ ligands and the retromer complex through binding to the VPS26 subunit (Gallon et al., 2014). It acts as an adaptor for several cargos, including G-protein-coupled receptors (GPCRs) such as the β2-adrenergic receptor (β2AR) and parathyroid hormone receptor (PTHR). It also interacts with proteins involved in neuronal plasticity, including α-amino-3-hydroxy-5-methyl-4-isoxazolepropionic acid receptors (AMPARs), NMDA receptors (NMDARs) and 5-hydroxytryptamine 4a receptors (5-HT4(a)Rs), and glucose transporter 1 (GLUT1) etc. during their transport from endosomes to the cell surface (Chan et al., 2016; Hussain et al., 2014; Joubert et al., 2004; Lauffer et al., 2010; Seaman, 2004; Steinberg et al., 2013; Temkin et al., 2011). However, no regulatory mechanisms for SNX27-retromer assembly during receptor recycling have been reported. In this study, we identified that PTEN physically interacts with SNX27 and prevents its association with the retromer complex, hampering endosomal recycling of GLUT1 to the plasma membrane.

RESULTS

SNX27 Is a PTEN-Associated Protein

Recent studies have highlighted the importance of the C-terminal PDZ binding motif (TKV) of PTEN in co-ordinating protein

This is an open access article under the CC BY license (http://creativecommons.org/licenses/by/4.0/).

OPEN
ACCESS
CellPress

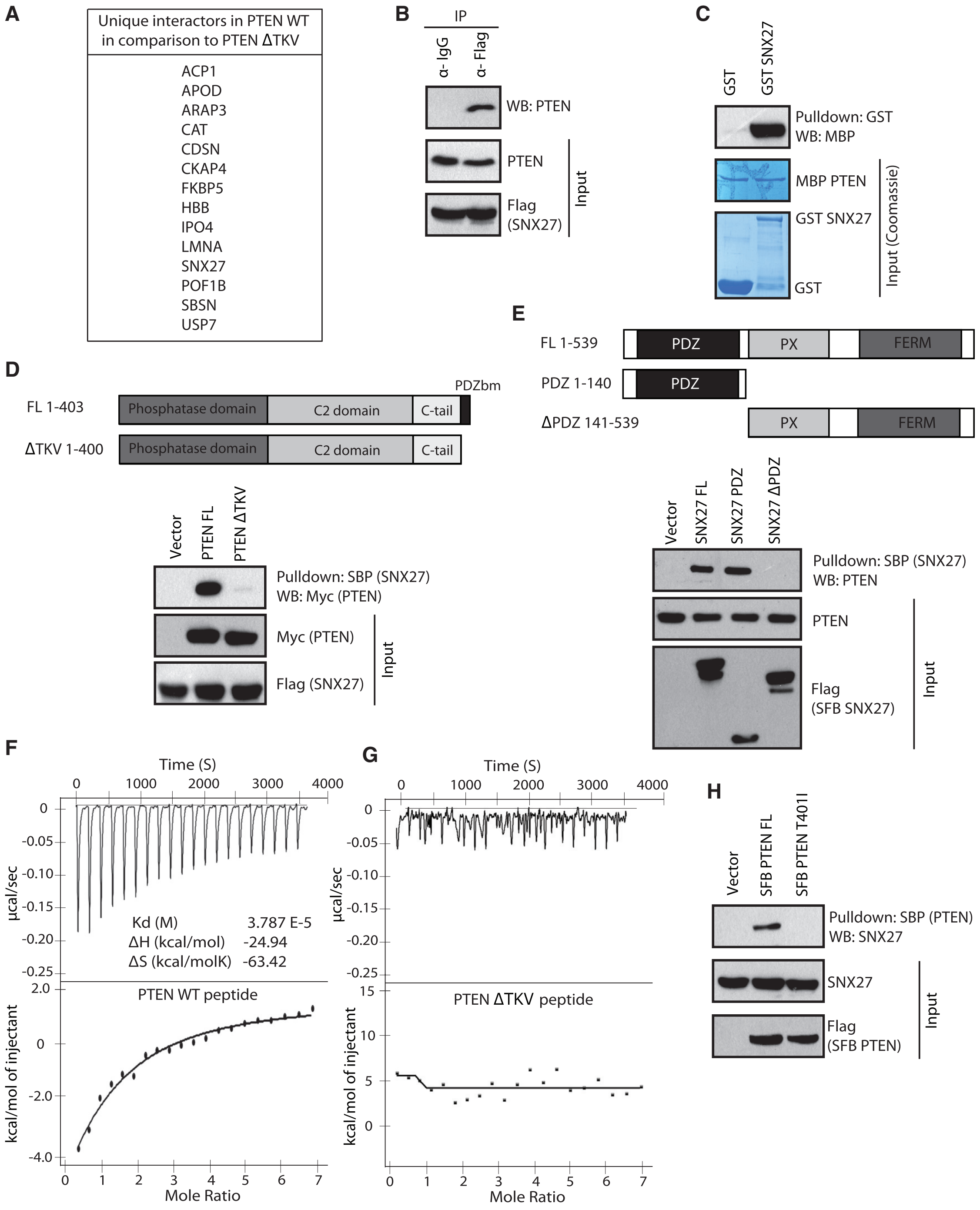

Figure 1. SNX27 Is a PTEN-Associated Protein

(A) List of unique interactors found in purification of PTEN wild-type (WT) after comparison with the purification list from the PTEN ΔTKV mutant, identified in proteomic analysis. The common interactors shared by both of them are excluded.

(B) HEK293T cells were transfected with SFB-tagged SNX27, and the cell lysates were subjected to immunoprecipitation (IP) with either control immunoglobulin G (IgG) or FLAG antibody, and the interaction of endogenous PTEN was determined by western blot (WB).

(C) Glutathione Sepharose beads immobilized with bacterially expressed recombinant GST or GST-SNX27 proteins were incubated with bacterially purified recombinant MBP-PTEN. The association of SNX27 with PTEN was detected by immunoblotting with MBP antibody. Expression of all recombinant proteins was shown by Coomassie staining.

(D) Schematic of N-terminal Myc-tagged versions of PTEN (full-length [FL]) and PTEN ΔTKV (top). Myc-tagged PTEN constructs and SFB-SNX27 were co-expressed in HEK293T cells, and the interaction of PTEN with SNX27 was detected by immunoblotting with anti-Myc antibodies after the cell lysates were pulled down with streptavidin beads (bottom).

(legend continued on next page)

complexes and tumor suppression (Takahashi et al., 2006; Valiente et al., 2005; van Ree et al., 2016). Thus, in an attempt to further unravel the cellular roles of the PTEN PDZ binding motif, we set out to perform a comparative interactome analysis using wild-type PTEN and a ΔTKV version of PTEN. The ΔTKV mutant of PTEN with intact secondary structure (Figure S1A) retains its phosphatase activity, similar to wild-type PTEN (Figure S1B). We identified protein complexes associated with full-length PTEN and the PTEN ΔTKV mutant by performing tandem affinity purification coupled with mass spectrometric identification. Our analysis has revealed several unique interactors in the wild-type PTEN list that were lost in ΔTKV mutant purification (Figure 1A). Among these interacting proteins, we found SNX27 (Sorting nexin 27), a PDZ domain-containing protein, to be a possible functionally relevant PTEN partner. The association of endogenous PTEN with SNX27 was confirmed by using immunoprecipitation (Figure 1B). Also, we found efficient association (Figure S1C) as well as co-localization (Figure S1D) between exogenously expressed PTEN and SNX27. Further, bacterially purified recombinant GST-SNX27 and maltose-binding protein (MBP)-PTEN proteins interacted with each other, suggesting a direct interaction between PTEN and SNX27 (Figure 1C). Because the C-terminal tail region of PTEN contains a class I PDZ binding motif and SNX27 has a PDZ domain in its structure, we hypothesized that PTEN and SNX27 may interact with each other through PDZ-PDZ binding motif association. In support of our hypothesis, we found that deletion of the PDZ binding motif (TKV) resulted in loss of PTEN interaction with SNX27 under *in vivo* (Figure 1D) as well as *in vitro* (Figure S1E) conditions. On the other hand, immunoprecipitation using the deletion mutants of SNX27 suggested that the PDZ domain of SNX27 is necessary and sufficient for its interaction with PTEN (Figure 1E; Figure S1F). Furthermore, by using isothermal titration calorimetry, we found that wild-type PTEN peptide binds with SNX27 (K_d of 37 μM) (Figure 1F); however, a peptide lacking the C-terminal PDZ binding motif shows no significant binding (Figure 1G). Interestingly, our search for PTEN somatic mutations outside its phosphatase domain using the Catalogue of Somatic Mutations in Cancer (COSMIC) database revealed a pathogenic PTEN mutation at the 401 position in the PDZ binding motif, where threonine is mutated to isoleucine in soft tissue sarcoma. The T401I mutant with intact secondary structure (Figure S2A) has phosphatase activity similar to wild-type PTEN (Figure S2B). Importantly, although full-length PTEN efficiently associates with SNX27, the T401I mutant is severely defective in binding to SNX27 (Figure 1H; Figure S2C), further supporting the importance of an intact PDZ binding motif in PTEN for their interaction. Together, these experiments demonstrate that PTEN binds to the PDZ domain of SNX27 through its C-terminal PDZ binding motif.

PTEN Prevents Endosome-to-Plasma Membrane Recycling of GLUT1

SNX27 functions in endosomal recycling of several membrane receptors, including GLUT1 (Steinberg et al., 2013), and it is well established that PTEN is critical for glucose homeostasis in the cell (Garcia-Cao et al., 2012). We therefore investigated whether PTEN-SNX27 association has a functional role in controlling glucose transport via the GLUT1 receptor. Depletion of PTEN in HeLa cells (Figure 2A) resulted in increased GLUT1 levels at the plasma membrane (Figure 2B). Similar observations were also made in HepG2 cells (Figures S3A–S3C). Further, co-depletion of SNX27 along with PTEN (Figure 2C) lowered GLUT1 at the plasma membrane, accumulated because of PTEN depletion alone (Figures 2D and 2E), suggesting that PTEN suppresses GLUT1 levels at the plasma membrane by controlling SNX27. Expression of short hairpin RNA (shRNA)-resistant full-length PTEN prevented the accumulation of membrane GLUT1 caused by PTEN depletion (Figures 2F and 2G). Intriguingly, the PTEN ΔTKV mutant, although it had intact phosphatase activity, failed to suppress accumulation of plasma membrane GLUT1, possibly indicating a phosphatase-independent function of PTEN in the regulation of GLUT1. In addition, although full-length PTEN reduces GLUT1 at the membrane, the PTEN T401I mutant fails to do so, again supporting the non-catalytic role of PTEN in the regulation of GLUT1. Suppression of membrane GLUT1 by wild-type PTEN but not the ΔTKV mutant was also observed in two independent cell lines, MDA-MB231 (Figures S3D and S3E) and MiaPaca2 (Figures S3F and S3G). To further test the possibility that PTEN reduces GLUT1 at the membrane by interfering with the SNX27 recycling function, we performed a colocalization analysis of GLUT1 with sorting endosomes. Expression of full-length PTEN, but not the ΔTKV mutant, led to a reduction in co-localization of GLUT1 with Rab5 (Figures 3A and 3B). Similarly, although full-length PTEN decreased GLUT1-Rab5 co-localization, the T401I mutant had no effect on them (Figures 3A and 3B). Furthermore, wild-type PTEN, but not the ΔTKV mutant or T401I mutant, reduced GLUT1 association with Rab11-positive recycling endosomes (Figures 3C and 3D). Defective sorting of GLUT1 to recycling endosomes because of PTEN expression may result in rerouting of GLUT1 to lysosomes. Indeed, we observed enhanced colocalization of GLUT1 with lysosomes in the presence of full-length PTEN. In contrast, expression of either the PTEN ΔTKV mutant or the T401I mutant showed no significant differences in GLUT1 localization at lysosomes compared with control cells (Figures 3E and 3F). Taken together, these data demonstrate that PTEN governs endosome-to-membrane trafficking of the SNX27-retromer cargo GLUT1.

(E) Schematic of N-terminal SFB-tagged SNX27 FL along with its deletion mutants (top). HEK293T cells were transfected with SFB-tagged SNX27 constructs, and the interaction of SNX27 with endogenous PTEN was detected by immunoblotting with anti-PTEN antibody after the cell lysates were pulled down with streptavidin beads (bottom).

(F and G) Wild-type PTEN peptide (DQHTQITKV) (F) and PTEN ΔTKV (DQHTQI) peptide (G) were titrated against full-length His-tagged SNX27 in ITC experiment.

(H) HEK293T cells were transfected with SFB-tagged wild-type FL PTEN or the T401I mutant, and the interaction with endogenous SNX27 was detected by immunoblotting with anti-SNX27 antibodies after the cell lysates were pulled down with streptavidin beads.

OPEN ACCESS
CellPress

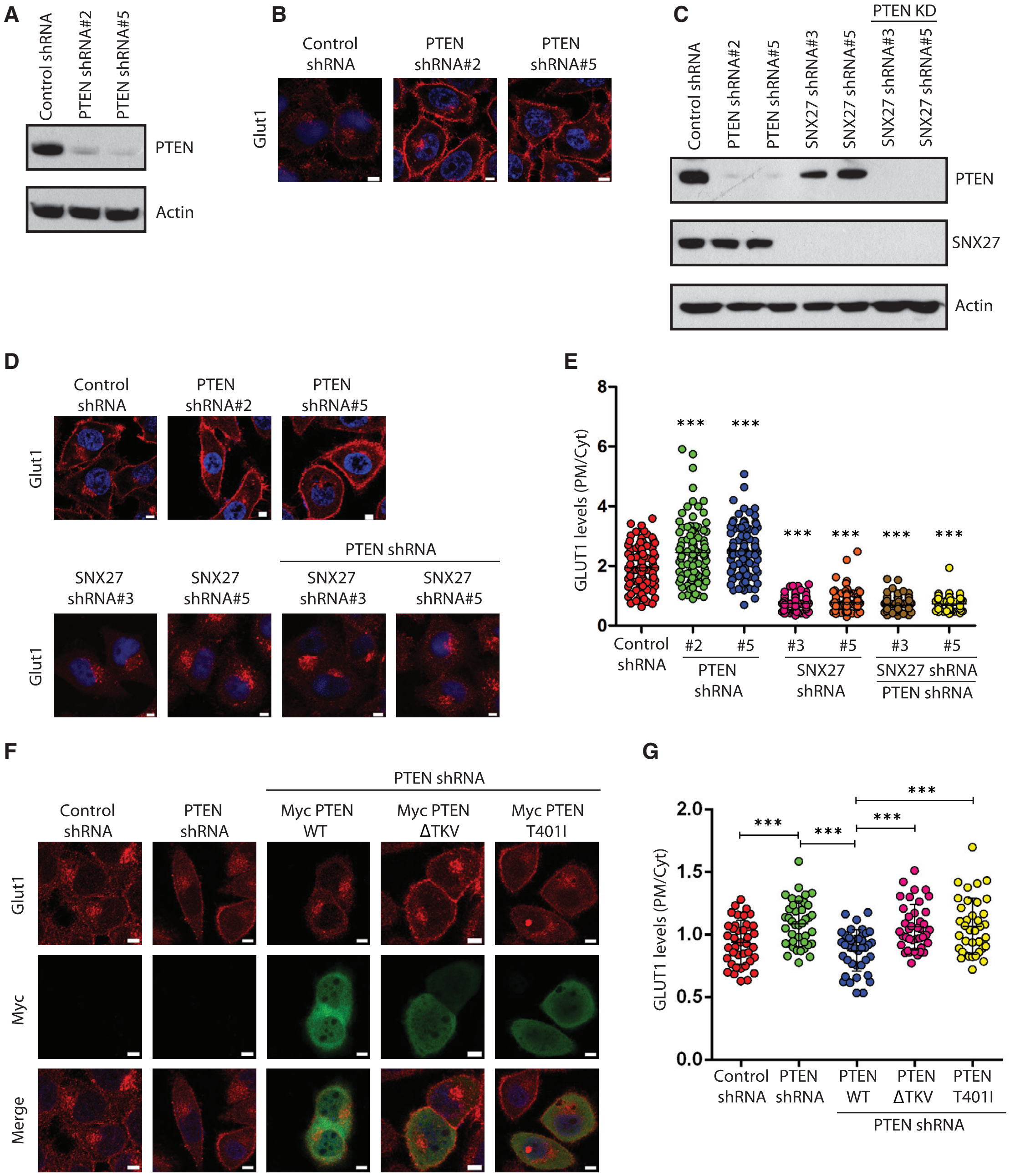

Figure 2. PTEN Prevents Endosome-to-Plasma Membrane Recycling of GLUT1.

(A) A representative western blot showing the shRNA-mediated knockdown of PTEN in HeLa cells.

(B) PTEN-depleted HeLa cells were fixed and imaged using a confocal microscope after staining with antibodies against GLUT1. Representative images are shown, Scale bars, 5 μm.

(C) HeLa cells were transduced with PTEN shRNAs alone or in combination with SNX27 shRNAs, and a representative western blot showing the knockdown of PTEN and SNX27 is shown.

(D) HeLa cells transduced with the indicated shRNAs were fixed and imaged using a confocal microscope after staining with antibodies against GLUT1. Representative images are shown. Scale bars, 5 μm.

(E) Quantification of plasma membrane (PM)-to-cytoplasmic (Cyt) ratio of GLUT1 intensity per each cell was plotted. Error bars indicate SD (n = 100 cells). ***$p < 0.001$ by Student's t test.

(F) HeLa cells transduced with PTEN shRNA were transfected with shRNA-resistant PTEN WT or the indicated mutants and then processed for staining with GLUT1. Representative images are shown. Scale bars, 5 μm.

(G) Quantification of PM-to-Cyt ratio of GLUT1 intensity per each cell was plotted. Error bars indicate SD (n = 40 cells). ***$p < 0.001$ by Student's t test.

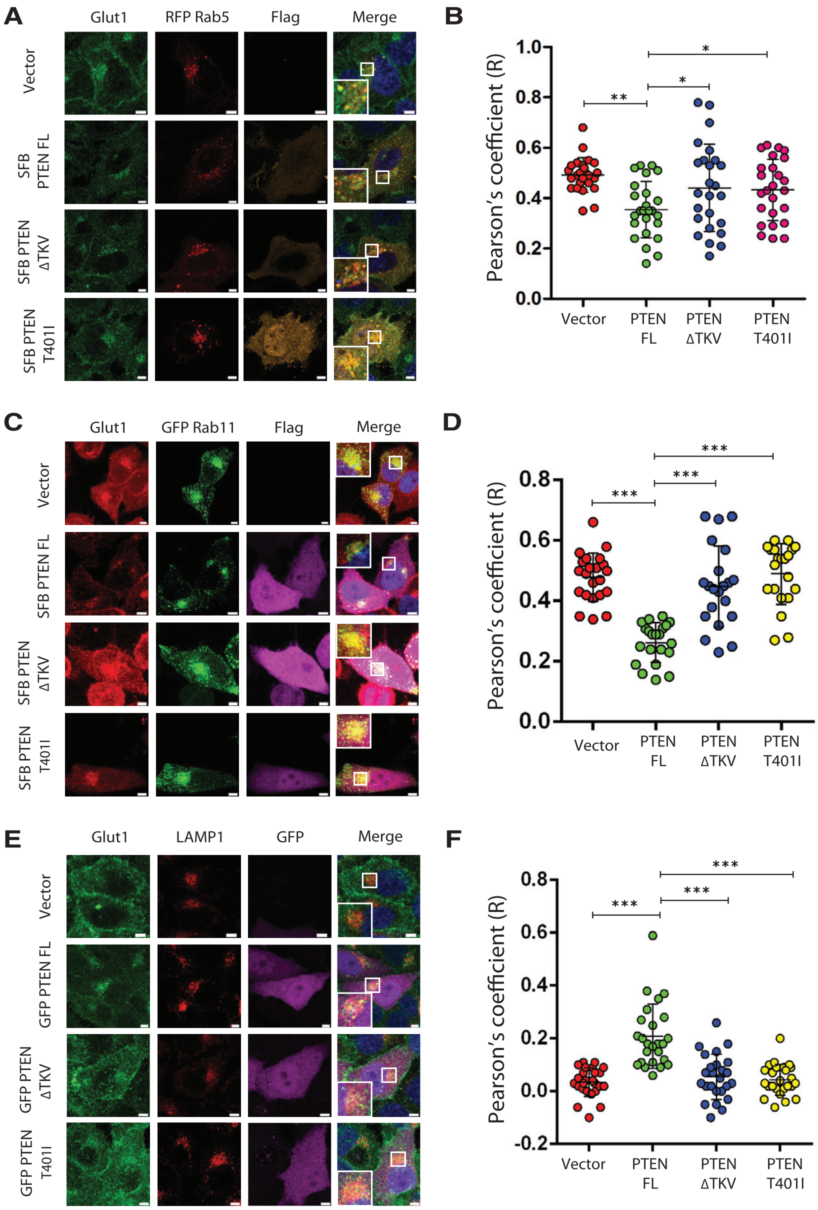

Figure 3. PTEN Reroutes GLUT1 to Lysosomes

(A) HeLa cells were transfected with SFB-tagged FL PTEN, ΔTKV, or the T401I mutant. The localization of GLUT1 at red fluorescent protein (RFP) Rab5-positive early endosomes was determined by staining with GLUT1 antibody. Scale bars, 5 μm.

(B) GLUT1 co-localization with RFP Rab5 was analyzed with the Pearson's correlation coefficient method of pixel intensity correlation measurements using Zeiss ZEN 2012 Blue Edition software. Error bars indicate SD (n = 25 expressing cells). *p < 0.5, **p < 0.01 by Student's t test.

(C) The localization of GLUT1 at GFP-Rab11-positive recycling endosomes in the presence of PTEN or its mutants was determined by staining with GLUT1 antibody. Scale bars, 5 μm.

(D) GLUT1 co-localization with GFP-Rab11 was analyzed. Error bars indicate SD (n = 25 expressing cells). *p < 0.5. **p < 0.01 by Student's t test.

(E) HeLa cells were transfected with SFB-PTEN full-length, ΔTKV, or the T401I mutant. Co-localization of GLUT1 with LAMP1 was determined by staining with the respective antibodies. Scale bars, 5 μm.

(F) GLUT1 co-localization with LAMP1 was analyzed with the Pearson's correlation coefficient method of pixel intensity correlation measurements using Zeiss ZEN 2012 Blue Edition software. Error bars indicate SD (n = 25 expressing cells). ***p < 0.001 by Student's t test.

PTEN Modulates Glucose Uptake in an SNX27-Dependent Manner

GLUT1 is widely expressed in almost all types of cells and tissues and is required for basal glucose uptake (Bogan, 2012; Szablewski, 2013). Dysregulation of GLUT1 leads to imbalanced glucose metabolism and contributes to the Warburg effect and tumorigenesis (Bentley et al., 2003; Feng and Levine, 2010; Ganapathy et al., 2009; Hanahan and Weinberg, 2011; Warburg, 1956). Because we observed that PTEN regulates membrane GLUT1 levels, we next tested the importance of PTEN-SNX27 association in glucose transport. As expected, depletion of PTEN (Figure 4A) significantly enhanced cellular uptake of glucose (Figure 4B). Similar observations were made in two other independent cell lines, HepG2 and Panc1 (Figures S4A and S4B). Additionally, a significant reduction in glucose uptake was detected in cells expressing shRNA-resistant full-length PTEN but not the PTEN ΔTKV or PTEN T401I mutant (Figure 4C). Previous studies have reported that Akt is required for PTEN-controlled GLUT1 expression and glucose uptake (Phadngam et al., 2016). However, the PTEN ΔTKV and PTEN T401I mutants, although they have intact phosphatase activity, could not fully repress glucose uptake. This possibly suggests that PTEN might have additional mechanisms to control GLUT1 membrane expression and glucose uptake besides the Akt pathway. Consistent with earlier studies, treatment of cells with an Akt inhibitor (MK-2206) caused a significant reduction in glucose uptake (Figures 4D and 4E). However, we observed increased glucose uptake upon PTEN depletion, even in the presence of an active Akt inhibitor, in multiple cell lines (Figures 4D and 4E and Figure S4C), fully supporting our conclusions that PTEN may function through additional pathways to control glucose transport independent of its phosphatase function and classical Akt pathway. To further test whether the alternative mechanism of PTEN-controlled glucose transport proceeds through the SNX27-dependent pathway, we performed double depletion experiments. Indeed, simultaneous depletion of SNX27 in the presence of PTEN shRNAs rescued the enhanced glucose uptake caused by PTEN depletion alone (Figure 4F). Overexpression of GLUT1, which results in increased glucose uptake, is often correlated with altered cellular metabolism and

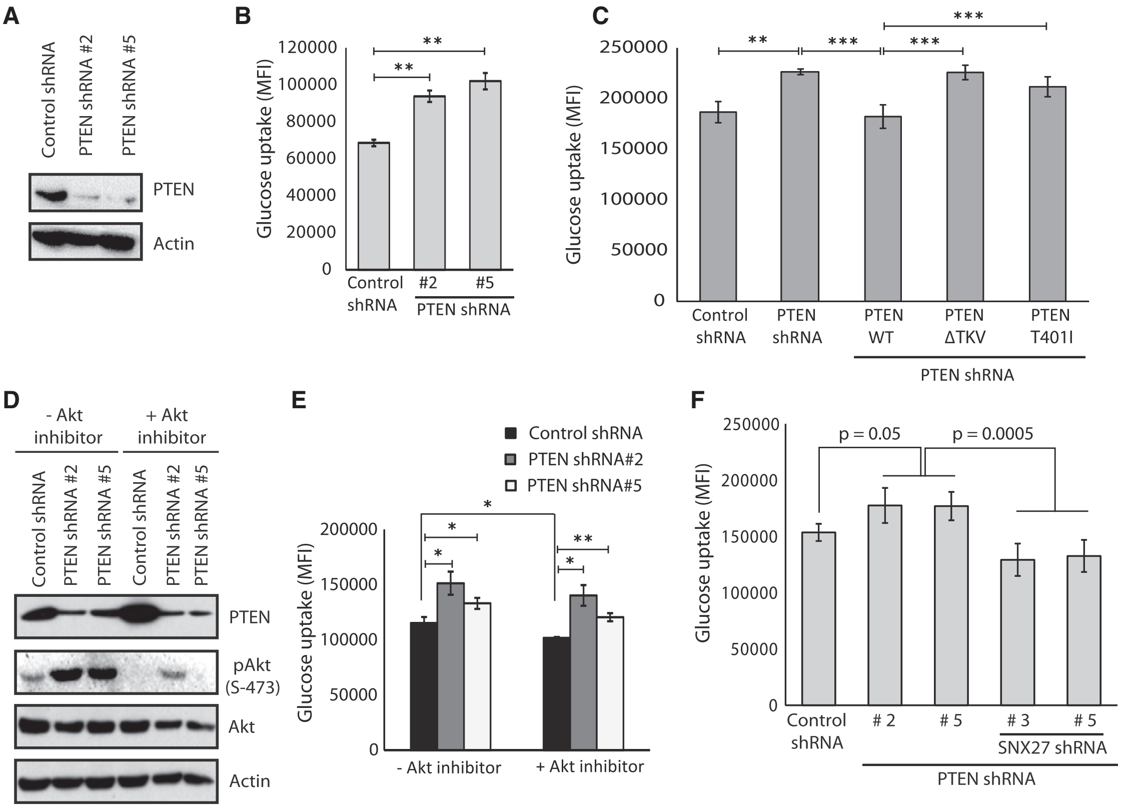

Figure 4. PTEN Modulates Glucose Uptake in an SNX27-Dependent Manner

(A) HeLa cells were transduced with either control or PTEN shRNAs and the protein levels of PTEN were assessed by WB.

(B) HeLa cells transfected with the indicated shRNAs were incubated with the fluorescent glucose analog 2-NBDG (100 μg/mL). Cellular uptake of glucose was measured by using a BD Accuri C6 flow cytometer. Data were acquired using BD Accuri C6 software and analyzed with FlowJo software. Data are expressed as MFI (median fluorescence intensity) from three independent experiments. Error bars indicate SD. **$p < 0.01$ by Student's t test.

(C) HeLa cells transduced with PTEN shRNA were transfected with shRNA-resistant PTEN WT or the indicated mutants, and then glucose uptake was measured. Error bars indicate SD. **$p < 0.01$, ***$p < 0.001$ by Student's t test.

(D) HeLa cells transduced with either control or PTEN shRNAs were treated with an Akt inhibitor (MK-2206, 10 μM) for 2 hr, and the cell lysates were subjected to SDS-PAGE analysis. The proteins were detected by immunoblotting with specific antibodies.

(E) Glucose uptake in these treated cells was measured. Error bars indicate SD. *$p < 0.5$, **$p < 0.01$ by Student's t test.

(F) HeLa cells were transduced with PTEN shRNAs alone or in combination with SNX27 shRNAs, and glucose uptake in these cells was measured. Error bars indicate SD. The p value was calculated by Student's t test.

the Warburg effect. To test this possibility, we measured intracellular lactate levels. We observed significantly increased lactate levels in multiple cancer cells upon PTEN depletion (Figure S4D). Conversely, a significant reduction in lactate levels was observed in cells expressing full-length PTEN but not the PTEN ΔTKV mutant (Figure S4E). Taken together, our data suggest that PTEN, through its PDZ binding motif, inhibits SNX27-retromer-mediated endosomal recycling of GLUT1 to the membrane, thereby governing glucose uptake. Further, increased lactate levels are suggestive of a probable role of the PTEN PDZ binding motif in suppressing the Warburg effect and tumorigenesis.

PTEN Inhibits SNX27-VPS26 Retromer Association Independent of Its Catalytic Activity

Next, we sought to gain mechanistic insight into PTEN-mediated regulation of the SNX27-GLUT1 pathway. SNX27 is known to bind to its cargo GLUT1 as well as a retromer component, VPS26, via its PDZ domain. Because we found that PTEN also binds to the PDZ domain of SNX27, we speculated that PTEN might inhibit the binding of either GLUT1 or VPS26. GLUT1 utilizes a canonical GYGF motif in the SNX27 PDZ domain (residues 50–53) for its binding. Although mutation of the SNX27 GYGF motif (F53A) abrogated GLUT1 binding, SNX27-PTEN interaction was unaffected (Figure S5A), possibly suggesting that PTEN does not share the same binding site as GLUT1. In fact, overexpression of PTEN has no effect on GLUT1 binding to SNX27 (Figure S5B). However, the association of VPS26 with SNX27 is severely hampered in the presence of PTEN (Figure 5A). Importantly, neither the ΔTKV version (Figure 5A) nor the T401I mutant of PTEN (Figure 5B) affected SNX27-VPS26 association, suggesting a critical role of the PTEN PDZ binding motif in regulating their interaction. Our *in vitro* competition experiments using bacterially purified recombinant proteins further suggested that only PTEN versions with an intact PDZ binding motif

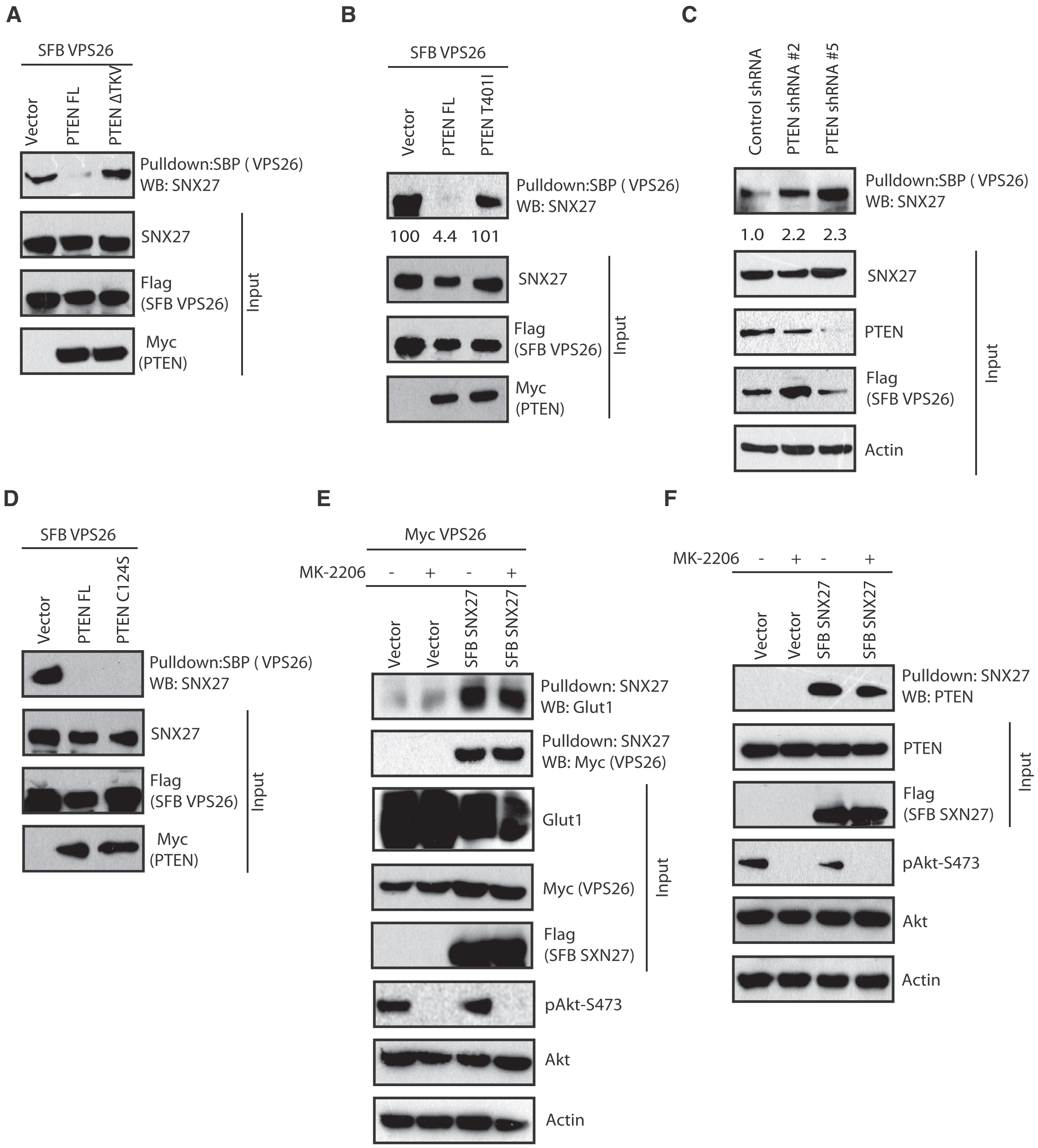

Figure 5. PTEN Disrupts SNX27-VPS26 Association Independent of Its Catalytic Activity

(A and B) SFB-tagged VPS26 along with Myc vector, Myc-tagged PTEN FL, or the PTEN ΔTKV mutant (A) or the PTEN T401I mutant (B) was co-transfected in HEK293T cells, and cell lysates were subjected to pull-down with streptavidin beads. The interaction of VPS26 with SNX27 was determined by western blotting with endogenous SNX27 antibody. The inputs were blotted with the respective antibodies.

(C) HeLa cells transduced with either control or PTEN shRNAs were transfected with SFB-tagged VPS26, and the cell lysates were subjected to pull-down with streptavidin beads. Interaction of VPS26 with SNX27 was determined by western blotting with endogenous SNX27 antibody.

(D) HEK293T cells were co-transfected with SFB-tagged VPS26 along with Myc- tagged PTEN FL or the catalytically inactive PTEN C124S mutant. Cell lysates were pulled down with streptavidin beads, and interaction was detected by immunoblotting with SNX27 antibodies.

(E) Cells were transfected with SFB vector or SFB-SNX27 along with Myc-VPS26. 24 hr after transfection, cells were treated with an Akt inhibitor (MK-2206, 10 μM) for 4 hr. Cells lysates were subjected to pull-down with streptavidin beads, and western blotting was performed using specific antibodies.

(F) Cells were transfected with SFB vector or SFB-SNX27 and treated with an Akt inhibitor. Cell lysates were subjected to pull-down with streptavidin beads, and endogenous interaction of PTEN was detected.

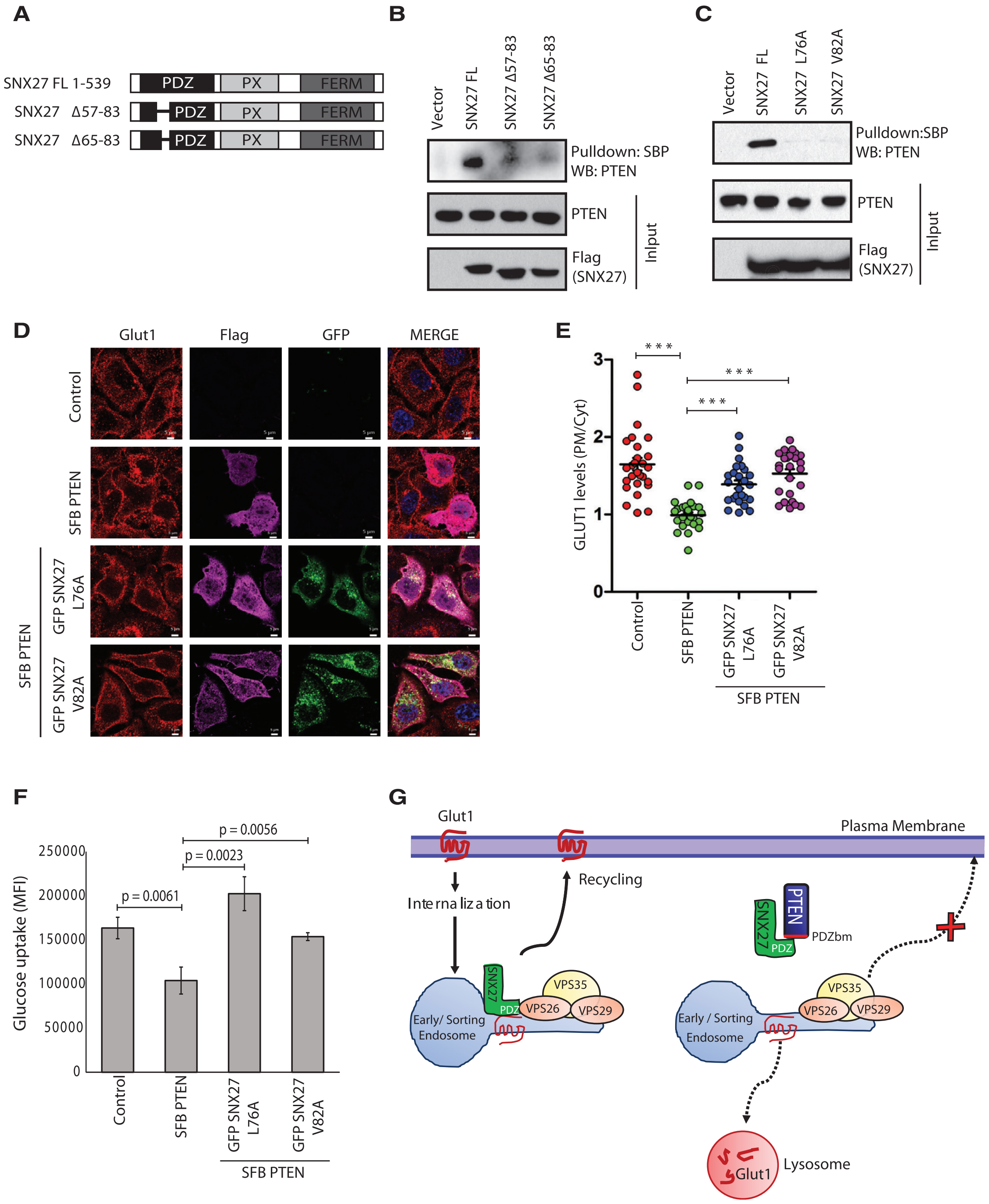

Figure 6. SNX27 Mutants Rescue PTEN-Mediated Repression of GLUT1 Levels and Glucose Uptake

(A) Schematic of N-terminal SFB-tagged SNX27 FL and the SNX27 Δ57–83 and SNX27 Δ65–83 mutants.

(B) The indicated constructs were expressed in HEK293T cells, and the interaction of PTEN with SNX27 was detected by immunoblotting with endogenous PTEN antibody after the cell lysates were pulled down with streptavidin beads.

(C) HEK293T cells were transfected with the SFB-tagged SNX27 FL or the SNX27 L76A or SNX27 V82A mutants, and cell lysates were subjected to pull-down with streptavidin beads. The interaction of SNX27 with PTEN was determined by western blotting with PTEN antibody.

(D) HeLa cells were transfected with SFB-tagged PTEN alone or co-transfected along with GFP-tagged versions of the SNX27 L76A and V82A mutants, and their effect on GLUT1 membrane levels was determined by confocal imaging after co-staining with FLAG and GLUT1 antibodies. Representative images are shown, Scale bars, 5 μm.

(E) Quantification of PM-to-Cyt ratio for GLUT1 intensity per each cell was plotted. Error bars indicate SD (n = 25 cells). ***$p < 0.001$ by Student's t test.

(legend continued on next page)

could reduce the binding between SNX27 and VPS26 (Figure S5C). In a control experiment, another PDZ domain-containing phosphatase, PTPN3, had no effect on SNX27-VPS26 interaction. Furthermore, a synthetic PTEN peptide with intact C-terminal TKV residues could compete with VPS26 binding with SNX27 (Figure S5D). Additionally, we observed significantly increased association of SNX27 and VPS26 upon PTEN depletion (Figure 5C). Similar to wild-type PTEN, overexpression of catalytically inactive PTEN (C124S mutant) also resulted in decreased SNX27-VPS26 association, suggesting that PTEN affects SNX27-retromer association independent of its catalytic activity (Figure 5D). Also, inhibition of Akt by MK-2206 did not affect the SNX27-GLUT1, SNX27-VPS26 (Figure 5E), or SNX27-PTEN association (Figure 5F), suggesting that the PTEN-controlled SNX27-VPS26 pathway functions in an Akt-independent manner. Together, these results demonstrate that PTEN, independent of its catalytic activity, inhibits SNX27-VPS26 retromer assembly.

Next, we sought to delineate the molecular details of how PTEN disrupts SNX27-VPS26 association. Structural analysis of the SNX27 PDZ domain bound to VPS26 has demonstrated that VPS26 binds to the conserved stretch of amino acids 64–74 between the βB-βC loop of the SNX27 PDZ domain (Gallon et al., 2014). To test the possibility that PTEN shares the same interaction surface on SNX27 and, thereby, blocks VPS26, we deleted amino acids 65–75 of the SNX27 PDZ domain (Figure S6A) and tested its interaction with PTEN. We observed that PTEN could still readily interact with the SNX27 Δ65–75 mutant (Figure S6B). It might be possible that PTEN has an alternate interaction surface on the SNX27 PDZ domain adjacent to the VPS26 binding region that might still hinder the association of VPS26 with SNX27. To further characterize the PTEN-SNX27 interacting region, we generated Δ57–83 and Δ65–83 deletion mutants of SNX27 (Figure 6A) and tested their interaction with PTEN. Although full-length SNX27 interacted with PTEN, the deletion mutants failed to do so (Figure 6B). Loss of PTEN interaction with these mutants may not be due to a misfolded PDZ domain in SNX27 because these deletion mutants show intact secondary structure (Figure S6C) and retain their ability to bind GLUT1 (Figure S6D). Overall, our immunoprecipitation data using various SNX27 deletion mutants suggested that the amino acid stretch of 75–83 of the SNX27 PDZ domain might be essential for interaction with PTEN. Further, to identify the key residues required for PTEN-SNX27 interaction, we mutated individual amino acids in this stretch on SNX27, located adjacent to the conserved VPS26 binding site (Figure S7A). Mutation of Leu 76 and Val 82 to alanine resulted in loss of PTEN-SNX27 interaction (Figure 6C; Figure S7B). However, these mutants could still readily interact (Figure S7C) and co-localize with VPS26 (Figure S7D) and GLUT1 (Figure S7E). Because we observed that SNX27 mutants failed to associate with PTEN but have intact binding with VPS26, we tested their ability to rescue PTEN-mediated reduction of GLUT1 levels and glucose uptake. Intriguingly, overexpression of SNX27 L76A and V82A mutants could rescue the GLUT1 levels at membrane (Figures 6D and 6E) as well as glucose uptake in PTEN-overexpressed cells (Figure 6F). These results suggest that PTEN binds with SNX27 adjacent to the VPS26 binding site and, therefore, restricts access to VPS26, leading to defective retromer assembly.

DISCUSSION

In conclusion, our data clearly demonstrate that PTEN binds with SNX27, hinders its access to the VPS26 retromer complex, and prevents the recycling of GLUT1 to the plasma membrane, followed by impaired glucose uptake (shown in the model in Figure 6G). A recent study (Garcia-Cao et al., 2012) has shown that elevated expression of PTEN in mice induces a tumor-suppressive metabolic state where less glucose is taken up, resembling an anti-Warburg state. Interestingly, they observed that PTEN induces this tumor suppressive metabolic state through PI3K-dependent as well as independent routes. Our current study linking PTEN with the SNX27 retromer pathway might fit one of the PI3K-independent mechanisms during PTEN-controlled glucose metabolism. We provided several lines of evidence to establish the role of the PTEN PDZ binding motif in the functional regulation of the SNX27-retromer complex. Recently, using engineered mice lacking the PTEN PDZ binding motif, it was shown that loss of the PTEN PDZ binding motif promotes aneuploidy and tumor formation (van Ree et al., 2016). Our studies further support the significant role of non-catalytic PTEN domains in normal physiological processes and tumorigenesis. Loss of the PDZ binding motif or mutations in this domain might alter cellular glucose homeostasis, resulting in high cellular proliferation and malignancy. Thus, our study provides a probable alternative mechanism for tumorigenesis in cancers with a truncated form of PTEN that lacks the PDZ binding motif, even though it has intact catalytic activity.

The SNX27-retromer complex participates in recycling of several cargos, such as GPCRs, β2AR, PTHR, AMPARs, NMDARs, lipoprotein receptor-related protein (LRP) receptors, and very low-density lipoprotein (VLDL) receptors, which are relevant to diverse cellular processes such as neurologic function, fat metabolism, and metal ion transport. Notably, PTEN was implicated to have a defining role in these distinct processes (Song et al., 2012). Thus, it is likely that PTEN might participate in recycling of some these receptors, possibly the cargoes that harbor PDZ ligands, which remains to be determined in future studies. Recently, phosphorylation of a subset of receptor cargos was shown to enhance their affinity for SNX27 binding (Clairfeuille et al., 2016). It is unlikely that PTEN has a direct role in dephosphorylating these receptor cargos because none of them were found to associate with PTEN in our interaction

(F) HEK293T cells were transfected with SFB PTEN alone or co-transfected along with FLAG-tagged versions of the SNX27 L76A and V82A mutants. Glucose uptake in these cells was measured. Error bars indicate SD. The p values were calculated by Student's t test.

(G) A proposed model for PTEN-mediated regulation of the SNX27-retromer complex during GLUT1 recycling. SNX27 recycles internalized GLUT1 from endosomes to the plasma membrane by linking PDZ-dependent cargo recognition to retromer-mediated transport. PTEN binds to the PDZ domain of SNX27 and disrupts its binding with VPS26 in the retromer complex, leading to the lysosomal degradation of GLUT1.

studies. However, it is possible that PTEN regulates trafficking of distinct cargos in phosphatase-dependent as well as -independent routes, depending on the cellular and signaling context, which needs to be tested in future studies. Our earlier study demonstrated that PTEN has a critical role in epidermal growth factor receptor (EGFR) trafficking and late endosome maturation through its protein phosphatase activity (Shinde and Maddika, 2016), and in the current study we documented the phosphatase-independent PTEN role in regulating endosomal recycling of GLUT1. This suggests PTEN as a mechanistically diverse regulator of endocytic trafficking, combining versatile phosphatase-dependent functions with a phosphatase-independent role that ensures homeostasis of signaling receptors in the cell.

EXPERIMENTAL PROCEDURES

Plasmids

Full-length PTEN as well as various PTEN deletion mutants were cloned into Myc-, glutathione S-transferase (GST)-, His-, MBP-, and SFB (S-protein/FLAG/SBP)-tagged destination vectors using the Gateway cloning system (Invitrogen). The point mutants of PTEN were generated by PCR-based site-directed mutagenesis and cloned into MBP-, SFB-, and Myc-tagged destination vectors. The mammalian cDNA for SNX27 and VPS26 was a kind gift from Dr. Peter J. Cullen (University of Bristol, Bristol, UK). Full-length and various deletion mutants of SNX27 were cloned into mammalian and bacterial expression vectors using the Gateway cloning system. The point mutations for SNX27 and shRNA-resistant PTEN variants were generated by PCR-based site-directed mutagenesis and cloned into various tagged destination vectors. VPS26 was cloned into SFB, Myc, and His destination vectors. mRFP Rab5 was kindly provided by Dr. Marino Zerial (Max Planck Institute of Molecular Cell Biology and Genetics, Germany). GFP-Rab11 was a kind gift from Dr. Mahak Sharma (Indian Institute of Science Education and Research [IISER] Mohali, India).

Antibodies and Reagents

The following commercial antibodies were used in this study: anti-PTEN (1:1,000, western blot [WB]), anti-pAkt (ser 473) (1:1,000, western blot), and anti-Akt (1:1,000, western blot) (Cell Signaling Technology); anti-SNX27 (1:100, immunofluorescence [IF]; 1:1,000, western blot), anti-GLUT1 (1:100, IF), and anti-LAMP1 (1:50, IF) (Abcam); anti-Myc (9E10) (1:1,000, western blot; 1:100, IF), anti-GFP (1:1,000, western blot), and anti-GST (1:5,000, western blot) (Santa Cruz Biotechnology); and anti-MBP (1:10,000, western blot), anti-FLAG (1:10,000, western blot; 1:200, IF), and anti-actin (1:10,000, western blot) (Sigma). Horseradish peroxidase (HRP)conjugated anti-mouse and anti-rabbit secondary antibodies were obtained from Jackson Immunological. The 2-(N-(7-Nitrobenz-2-oxa-1,3-diazol-4-yl)Amino)-2-Deoxyglucose (2-NBDG)-based glucose uptake assay kit (Cayman Chemical) and lactate calorimetric assay kit (Bio Vision Technologies) were used in the study. Synthetic peptides with the PTEN sequence were purchased from GenScript.

Cell Lines and Transfection

HEK293T, HeLa, BOSC23, MDA-MB-231, HepG2, Panc1, and MiaPaca2 cell lines were used in this study. All cell lines were maintained in RPMI medium containing 10% fetal bovine serum (FBS) and penicillin and streptomycin (1%). Cells were continuously monitored by microscopy to maintain their original morphology and also tested for mycoplasma contamination by using DAPI staining. All cell lines were transfected with various plasmids using polyethylenimine (PEI) (Polysciences) or Turbofect (Invitrogen) according to the protocol of the manufacturer. MiaPaca2 cells were kindly provided by Dr. Murali Bashyam (Centre for DNA Fingerprinting and Diagnostics, Hyderabad, India).

Tandem Affinity Purification

HEK293T cells expressing S-protein/FLAG/SBP (streptavidin binding protein) triple-tagged full-length PTEN or the PTEN ΔTKV mutant were lysed with NETN buffer (20 mM Tris-HCl [pH 8.0], 100 mM NaCl, 1 mM EDTA, and 0.5% Nonidet P-40) containing 1 μg/mL each of pepstatin A and aprotinin on ice for 30 min. The cell debris was removed by centrifugation at 13,000 rpm for 10 min at 4°C. The cell lysates were incubated with streptavidin Sepharose beads (Amersham Biosciences) for 1 hr at 4°C. The bead-bound protein complexes were washed three times with NETN and then eluted with 2 mg/mL biotin (Sigma) for 1 hr at 4°C. The eluates were incubated with S-protein agarose beads (Novagen) for 1 hr at 4°C and then washed three times with NETN at 2,000 rpm for 2 min at 4°C. The proteins bound to S-protein agarose beads were eluted by boiling in SDS loading buffer and resolved by SDS-PAGE. The proteins were identified by mass spectrometry analysis (Taplin Biological Mass Spectrometry Facility at Harvard University). Purification of SFB-GFP was also performed in a similar way and was used as a control for removing the non-specific interacting proteins.

Immunoprecipitation and Western Blotting

For immunoprecipitation assays, cells were lysed with NETN buffer (20 mmol/L Tris-HCl [pH 8.0], 100 mmol/L NaCl, 1 mmol/L EDTA, and 0.5% Nonidet P-40) containing 50 mmol/L b-glycerophosphate, 1 mg/mL each of pepstatin A and aprotinin on ice for 20 min. The whole-cell lysates were incubated with either antibody-bound protein A or protein G Sepharose beads (Amersham Biosciences) or streptavidin beads for 1 hr at 4°C. The immunocomplexes were then washed with NETN buffer three times and eluted in SDS sample buffer. The proteins were separated by denaturing SDS-PAGE and then transferred onto polyvinylidene fluoride (PVDF) membranes. The membranes were blocked in 5% non-fat dried milk in Tris-buffered saline (TBS) for 30 min and then incubated overnight with the primary antibodies at 4°C. Then the blots were incubated with the corresponding secondary antibodies conjugated with HRP at room temperature for 1 hr. Visualization was carried out by enhanced chemiluminescence (ECL) detection (Thermo Fisher Scientific).

Recombinant Protein Purification

GST-tagged SNX27 full-length and mutants or MBP-tagged PTEN full-length and mutants or $(His)_6$-PTEN, SNX27, and VPS26 were transformed into *Escherichia coli* BL21 DE3 cells. Protein expression was induced with 0.5 mM isopropyl β-D-1-thiogalactopyranoside (IPTG) at 18°C overnight (for PTEN, 0.2 mM IPTG at 25°C for 6 hr). The cell pellet was resuspended in lysis buffer (50 mM Tris [pH 7.5], 150 mM NaCl, and 0.01% NP-40 Igepal and protease inhibitors) (for His-tagged proteins: 50 mM NaH_2PO_4 [pH 8.0], 300 mM NaCl, 20 mM imidazole, 10% glycerol, 0.01% Igepal, and protease inhibitors) and subjected to sonication. Cell lysates were incubated with either glutathione Sepharose (for GST-tagged proteins) or dextran Sepharose beads (for MBP-tagged proteins) or cobalt-based Talon beads (for His-tagged proteins) for 2 hr at 4°C. Beads were washed 5 times with wash buffer (50 mM Tris [pH 7.5], 300 mM NaCl, 0.01% NP-40 Igepal, 1 mM DTT, and protease inhibitors). The bound proteins were eluted with the elution buffer containing 50 mM NaH_2PO_4 (pH 8.0), 300 mM NaCl, and 300 mM imidazole (for His-tagged proteins) or 50 mM Tris (pH 8), 150 mM NaCl, and 10 mM reduced Glutathione (for GST-tagged proteins) or 20 mM Tris (pH 7.5), 200 mM NaCl, 1 mM EDTA, and 10 mM maltose (MBP-tagged proteins) for 30 min at 4°C.

In Vitro Binding Assays

Bacterially expressed GST-SNX27 or control GST bound to glutathione Sepharose beads (Amersham) was incubated with bacterial cell lysates expressing MBP PTEN for 2 hr at 4°C. The beads were washed three times with wash buffer and eluted by boiling in SDS sample buffer. The proteins were separated by SDS-PAGE, and the interactions were analyzed by western blotting. Similarly, bacterially expressed MBP-tagged PTEN bound to dextrose beads or His-tagged PTEN bound to Ni-NTA beads was incubated with bacterial cell lysates expressing GST-tagged SNX27 full-length or other mutants at 4°C for 2 hr. The interactions were detected by western botting.

In Vitro Competition Assays

Bacterially purified His-tagged VPS26 bound to Ni-NTA beads was incubated with 5 μM of GST-tagged full-length SNX27 along with increasing concentrations of MBP PTEN full-length or various mutants in buffer containing 50 mM Tris (pH 7.5), 150 mM NaCl, and protease inhibitors. Similarly, for peptide

competition assays, His-tagged VPS26 bound to Ni-NTA beads was incubated with 5 μM of GST-tagged full-length SNX27 along with different concentrations of PTEN peptides. After 2 hr of incubation at 4°C, the beads were washed four times with wash buffer, bound proteins were eluted in SDS-PAGE sample buffer, and western blot analysis was performed.

Isothermal Titration Calorimetry

His-tagged full-length SNX27 was purified and subjected to salt exchange on PD-10 columns into isothermal titration calorimetry (ITC) buffer (50 mM Tris [pH 8] and 100 mM NaCl). The synthetic PTEN peptide was purchased from GenScript. ITC experiments were performed on a NanoITC instrument in ITC buffer. Peptides (300 μM) were titrated into 15 μM SNX27 protein solution at 25°C. Data were processed using NanoAnalyze software to extract the thermodynamic parameters.

Secondary Structure Analysis by Circular Dichroism

His-tagged PTEN full-length or mutants and SNX27 full-length or mutants was purified, and the secondary structure conformational stability and integrity were assessed by circular dichroism (CD). Each spectrum, recorded from 190 nm to 260 nm, was an average of three accumulations. The data points accumulated in millidegree were converted to mean residue ellipticity (expressed in deg cm^2 $dmol^{-1}$ [θ]).

RNAi and Lentiviral Infection

Lentivirus-based PTEN and SNX27 shRNA (clones purchased from Open Biosystems) containing plasmids were transfected transiently using PEI (Invitrogen) in BOSC23 packaging cells along with packaging vectors. The viral medium was collected 48 hr after transfection and added to the target cells along with Polybrene (8 μg/mL). 48 hr after transduction, cells were collected and processed for various assays, and immunoblotting was performed with specific antibodies to check the efficiency of knockdown.

Immunofluorescence Staining

Cells were seeded in six-well plates containing coverslips. 24 hr after transfection, cells grown on coverslips were fixed with 3% paraformaldehyde solution in PBS containing 50 mM sucrose at room temperature for 15 min. After permeabilization with 0.5% Triton X-100 buffer containing 20 mM 4-(2-hydroxyethyl)-1-piperazineethanesulfonic acid (HEPES) at pH 7.4, 50 mM NaCl, 3 mM $MgCl_2$, and 300 mM sucrose at room temperature for 5 min, cells were incubated with 1% BSA for blocking at room temperature for 60 min. Cells were washed three times with 1× PBS and incubated with primary antibodies for 1 hr at room temperature. Further, the cells were subjected to a 1× PBS wash (3 times for 10 min each). Then cells were incubated with fluorescein isothiocyanate (FITC) or Rhodamine-conjugated secondary antibodies at room temperature for 60 min, followed by 3 washes with 1× PBS for 10 min each. Nuclei were counterstained with DAPI. After a final wash with PBS, coverslips were mounted with glycerine containing paraphenylenediamine. Images were taken using a confocal microscope (LSM Meta 510, Zeiss). z stacks with a 0.5-μm step size were acquired over a total imaging distance of 15 μm. To quantify colocalization, Pearson's correlation coefficient was measured using Zen software (Zeiss).

Measurement of GLUT1 Levels at the Plasma Membrane

Cells were stained with GLUT1 antibody and imaged using a Zeiss 700 confocal microscope. The GLUT1 levels at the plasma membrane were calculated using the following method. A line was drawn across the cell, and the mean intensity was measured for a 5-nm area at two independent sites of the cell surface. The mean of these two values was taken as the plasma membrane (PM) fraction, whereas the mean intensity of other area was taken as the cytoplasmic fraction (Cyt). Further, the ratio of cytoplasm to plasma membrane was calculated and plotted.

Measurements of Glucose Uptake in Cells

Cells were seeded in culture plates and cultured for 48 hr after virus infection or transfection. After 48 hr, cells were incubated with the fluorescent glucose analog 2-NBDG (in green, 100 μg/mL) for 1 hr. At the end of the treatment, cells were washed with 1× PBS, followed by a wash with cell-based assay buffer (according to the Cayman kit protocol). Cells were analyzed using a BD Accuri C6 flow cytometer. Data were acquired using BD Accuri C6 software (5,000 gated events/sample on FL-1) and analyzed with FlowJo software.

Measurements of Lactate Production in Cells

Cells were seeded in culture plates and cultured for 48 hr after virus infection or transfection. Intracellular lactate concentration was measured using the colorimetry-based lactate assay kit (BioVis). The lactate concentration was estimated from the standard curve and plotted.

Statistics

All data were obtained from three independent experiments. Data are reported as mean ± SD. Statistical analyses were carried out using GraphPad Prism. Student's t test was used to determine the statistical significance, where the differences were considered significant at a p value of less than 0.05 (***$p < 0.001$, **$p < 0.01$, or *$p < 0.05$).

SUPPLEMENTAL INFORMATION

Supplemental Information includes seven figures and can be found with this article online at https://doi.org/10.1016/j.celrep.2017.10.053.

AUTHOR CONTRIBUTIONS

S.M. conceptualized and managed the project. S.M. and S.R.S. designed the experiments, analyzed the data, and wrote the manuscript. S.R.S. performed all experiments.

ACKNOWLEDGMENTS

This work was fully supported by Wellcome Trust/DBT India Alliance grants (Intermediate Fellowship 500230/Z/11/Z and Senior Fellowship IA/S/16/2/502729 to S.M.). S.M. is a recipient of the Department of Biotechnology Senior IYBA award (BT/01/IYBA/2009). We thank Dr. Rohit Joshi's lab as well as the CDFD core facility for their assistance with confocal imaging. We thank the CDFD sophisticated equipment facility for their assistance with ITC as well as CD experiments. We also thank Dr. Sriyans Jain and Debasish Ghosh for their input regarding ITC and CD data. We thank Sawanth Suresh Kumar for providing important reagents. S.R.S. acknowledges the support of a senior research fellowship from the University Grants Commission, India. We thank all members of LCDCS for their critical input and Nanci Rani for providing technical assistance.

Received: June 9, 2017
Revised: September 19, 2017
Accepted: October 12, 2017
Published: November 7, 2017

REFERENCES

Ali, I.U., Schriml, L.M., and Dean, M. (1999). Mutational spectra of PTEN/MMAC1 gene: a tumor suppressor with lipid phosphatase activity. J. Natl. Cancer Inst. *91*, 1922–1932.

Bentley, J., Itchayanan, D., Barnes, K., McIntosh, E., Tang, X., Downes, C.P., Holman, G.D., Whetton, A.D., Owen-Lynch, P.J., and Baldwin, S.A. (2003). Interleukin-3-mediated cell survival signals include phosphatidylinositol 3-kinase-dependent translocation of the glucose transporter GLUT1 to the cell surface. J. Biol. Chem. *278*, 39337–39348.

Bogan, J.S. (2012). Regulation of glucose transporter translocation in health and diabetes. Annu. Rev. Biochem. *81*, 507–532.

Chan, A.S., Clairfeuille, T., Landao-Bassonga, E., Kinna, G., Ng, P.Y., Loo, L.S., Cheng, T.S., Zheng, M., Hong, W., Teasdale, R.D., et al. (2016). Sorting nexin 27 couples PTHR trafficking to retromer for signal regulation in osteoblasts during bone growth. Mol. Biol. Cell *27*, 1367–1382.

Clairfeuille, T., Mas, C., Chan, A.S., Yang, Z., Tello-Lafoz, M., Chandra, M., Widagdo, J., Kerr, M.C., Paul, B., Mérida, I., et al. (2016). A molecular code for

endosomal recycling of phosphorylated cargos by the SNX27-retromer complex. Nat. Struct. Mol. Biol. *23*, 921–932.

Di Cristofano, A., and Pandolfi, P.P. (2000). The multiple roles of PTEN in tumor suppression. Cell *100*, 387–390.

Duerr, E.M., Rollbrocker, B., Hayashi, Y., Peters, N., Meyer-Puttlitz, B., Louis, D.N., Schramm, J., Wiestler, O.D., Parsons, R., Eng, C., and von Deimling, A. (1998). PTEN mutations in gliomas and glioneuronal tumors. Oncogene *16*, 2259–2264.

Feng, Z., and Levine, A.J. (2010). The regulation of energy metabolism and the IGF-1/mTOR pathways by the p53 protein. Trends Cell Biol. *20*, 427–434.

Gallon, M., Clairfeuille, T., Steinberg, F., Mas, C., Ghai, R., Sessions, R.B., Teasdale, R.D., Collins, B.M., and Cullen, P.J. (2014). A unique PDZ domain and arrestin-like fold interaction reveals mechanistic details of endocytic recycling by SNX27-retromer. Proc. Natl. Acad. Sci. USA *111*, E3604–E3613.

Ganapathy, V., Thangaraju, M., and Prasad, P.D. (2009). Nutrient transporters in cancer: relevance to Warburg hypothesis and beyond. Pharmacol. Ther. *121*, 29–40.

Garcia-Cao, I., Song, M.S., Hobbs, R.M., Laurent, G., Giorgi, C., de Boer, V.C., Anastasiou, D., Ito, K., Sasaki, A.T., Rameh, L., et al. (2012). Systemic elevation of PTEN induces a tumor-suppressive metabolic state. Cell *149*, 49–62.

Gu, J., Tamura, M., Pankov, R., Danen, E.H., Takino, T., Matsumoto, K., and Yamada, K.M. (1999). Shc and FAK differentially regulate cell motility and directionality modulated by PTEN. J. Cell Biol. *146*, 389–403.

Hanahan, D., and Weinberg, R.A. (2011). Hallmarks of cancer: the next generation. Cell *144*, 646–674.

He, J., Zhang, Z., Ouyang, M., Yang, F., Hao, H., Lamb, K.L., Yang, J., Yin, Y., and Shen, W.H. (2016). PTEN regulates EG5 to control spindle architecture and chromosome congression during mitosis. Nat. Commun. *7*, 12355.

Hussain, N.K., Diering, G.H., Sole, J., Anggono, V., and Huganir, R.L. (2014). Sorting Nexin 27 regulates basal and activity-dependent trafficking of AMPARs. Proc. Natl. Acad. Sci. USA *111*, 11840–11845.

Joubert, L., Hanson, B., Barthet, G., Sebben, M., Claeysen, S., Hong, W., Marin, P., Dumuis, A., and Bockaert, J. (2004). New sorting nexin (SNX27) and NHERF specifically interact with the 5-HT4a receptor splice variant: roles in receptor targeting. J. Cell Sci. *117*, 5367–5379.

Lauffer, B.E., Melero, C., Temkin, P., Lei, C., Hong, W., Kortemme, T., and von Zastrow, M. (2010). SNX27 mediates PDZ-directed sorting from endosomes to the plasma membrane. J. Cell Biol. *190*, 565–574.

Li, J., Yen, C., Liaw, D., Podsypanina, K., Bose, S., Wang, S.I., Puc, J., Miliaresis, C., Rodgers, L., McCombie, R., et al. (1997). PTEN, a putative protein tyrosine phosphatase gene mutated in human brain, breast, and prostate cancer. Science *275*, 1943–1947.

Liaw, D., Marsh, D.J., Li, J., Dahia, P.L., Wang, S.I., Zheng, Z., Bose, S., Call, K.M., Tsou, H.C., Peacocke, M., et al. (1997). Germline mutations of the PTEN gene in Cowden disease, an inherited breast and thyroid cancer syndrome. Nat. Genet. *16*, 64–67.

Lunn, M.L., Nassirpour, R., Arrabit, C., Tan, J., McLeod, I., Arias, C.M., Sawchenko, P.E., Yates, J.R., 3rd, and Slesinger, P.A. (2007). A unique sorting nexin regulates trafficking of potassium channels via a PDZ domain interaction. Nat. Neurosci. *10*, 1249–1259.

Maehama, T., and Dixon, J.E. (1998). The tumor suppressor, PTEN/MMAC1, dephosphorylates the lipid second messenger, phosphatidylinositol 3,4,5-trisphosphate. J. Biol. Chem. *273*, 13375–13378.

Myers, M.P., Stolarov, J.P., Eng, C., Li, J., Wang, S.I., Wigler, M.H., Parsons, R., and Tonks, N.K. (1997). P-TEN, the tumor suppressor from human chromosome 10q23, is a dual-specificity phosphatase. Proc. Natl. Acad. Sci. USA *94*, 9052–9057.

Phadngam, S., Castiglioni, A., Ferraresi, A., Morani, F., Follo, C., and Isidoro, C. (2016). PTEN dephosphorylates AKT to prevent the expression of GLUT1 on plasmamembrane and to limit glucose consumption in cancer cells. Oncotarget *7*, 84999–85020.

Seaman, M.N. (2004). Cargo-selective endosomal sorting for retrieval to the Golgi requires retromer. J. Cell Biol. *165*, 111–122.

Shi, Y., Wang, J., Chandarlapaty, S., Cross, J., Thompson, C., Rosen, N., and Jiang, X. (2014). PTEN is a protein tyrosine phosphatase for IRS1. Nat. Struct. Mol. Biol. *21*, 522–527.

Shinde, S.R., and Maddika, S. (2016). PTEN modulates EGFR late endocytic trafficking and degradation by dephosphorylating Rab7. Nat. Commun. *7*, 10689.

Song, M.S., Salmena, L., and Pandolfi, P.P. (2012). The functions and regulation of the PTEN tumour suppressor. Nat. Rev. Mol. Cell Biol. *13*, 283–296.

Stambolic, V., Suzuki, A., de la Pompa, J.L., Brothers, G.M., Mirtsos, C., Sasaki, T., Ruland, J., Penninger, J.M., Siderovski, D.P., and Mak, T.W. (1998). Negative regulation of PKB/Akt-dependent cell survival by the tumor suppressor PTEN. Cell *95*, 29–39.

Steinberg, F., Gallon, M., Winfield, M., Thomas, E.C., Bell, A.J., Heesom, K.J., Tavaré, J.M., and Cullen, P.J. (2013). A global analysis of SNX27-retromer assembly and cargo specificity reveals a function in glucose and metal ion transport. Nat. Cell Biol. *15*, 461–471.

Szablewski, L. (2013). Expression of glucose transporters in cancers. Biochim. Biophys. Acta *1835*, 164–169.

Takahashi, Y., Morales, F.C., Kreimann, E.L., and Georgescu, M.M. (2006). PTEN tumor suppressor associates with NHERF proteins to attenuate PDGF receptor signaling. EMBO J. *25*, 910–920.

Temkin, P., Lauffer, B., Jäger, S., Cimermancic, P., Krogan, N.J., and von Zastrow, M. (2011). SNX27 mediates retromer tubule entry and endosome-to-plasma membrane trafficking of signalling receptors. Nat. Cell Biol. *13*, 715–721.

Valiente, M., Andrés-Pons, A., Gomar, B., Torres, J., Gil, A., Tapparel, C., Antonarakis, S.E., and Pulido, R. (2005). Binding of PTEN to specific PDZ domains contributes to PTEN protein stability and phosphorylation by microtubule-associated serine/threonine kinases. J. Biol. Chem. *280*, 28936–28943.

van Ree, J.H., Nam, H.J., Jeganathan, K.B., Kanakkanthara, A., and van Deursen, J.M. (2016). Pten regulates spindle pole movement through Dlg1-mediated recruitment of Eg5 to centrosomes. Nat. Cell Biol. *18*, 814–821.

Warburg, O. (1956). On the origin of cancer cells. Science *123*, 309–314.

Cell Reports, Volume *21*

Supplemental Information

PTEN Regulates Glucose Transporter Recycling by Impairing SNX27 Retromer Assembly

Swapnil Rohidas Shinde and Subbareddy Maddika

Figure S1

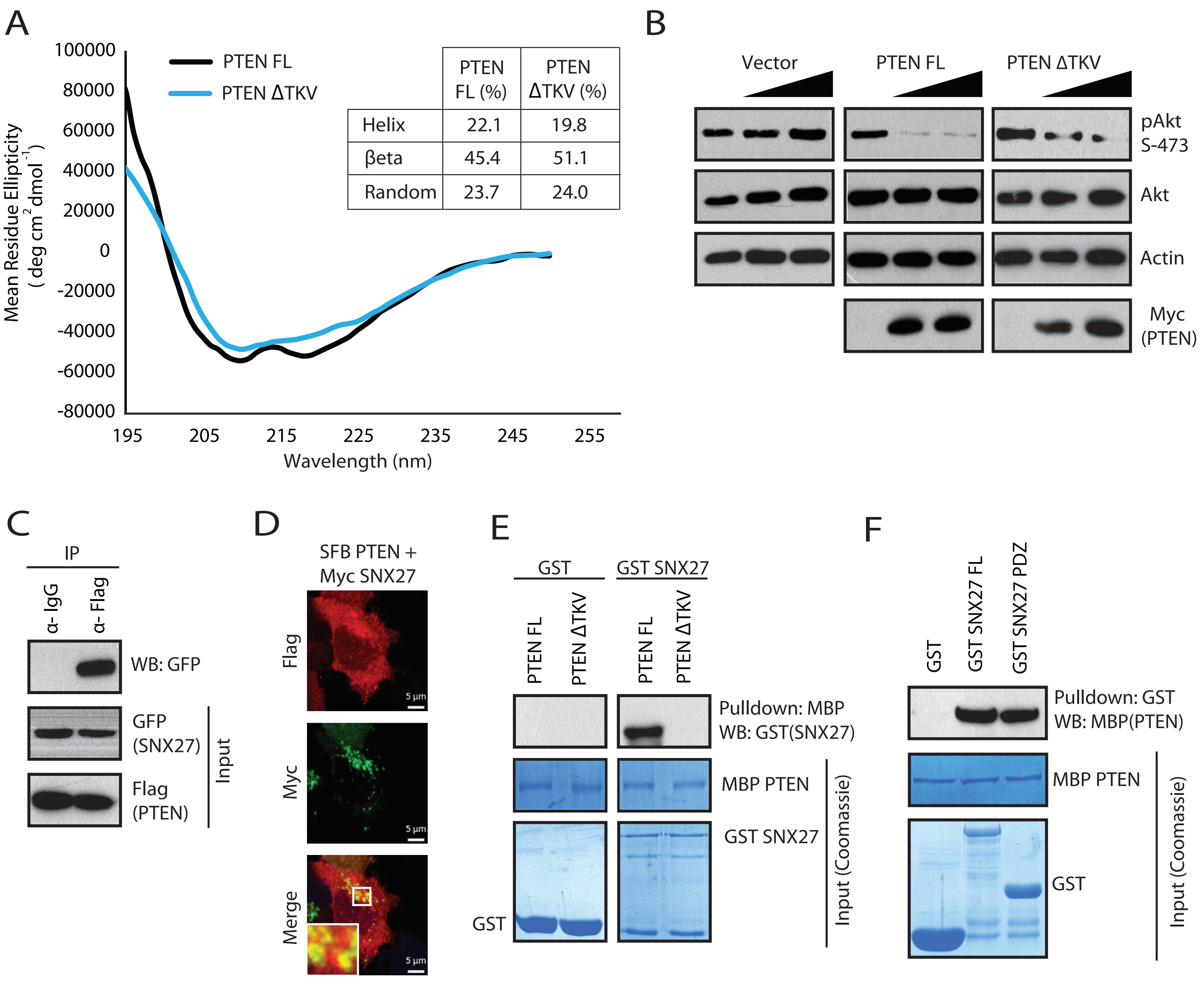

Figure S1, Related to Figure 1.
(A) The CD spectra for full length (FL) PTEN and ΔTKV mutant was shown. **(B)** MDA-MB-231 cells were transfected with vector, PTEN full length (FL) or PTEN ΔTKV mutant. Cell lysates were subjected to immunoblotting analysis and activation of Akt was detected by using a phospho-specific (ser 473) antibody. **(C)** HEK 293T cells were co-transfected with SFB-tagged PTEN along with GFP-SNX27 and the cell lysates were subjected to immunoprecipitation (IP) with either control IgG or Flag antibody and the interaction was determined by western blotting (WB) with GFP antibody. **(D)** Co-localization of exogenously expressed PTEN and SNX27 in cells was shown, scale bar 5 µm. **(E)** Agarose beads immobilized with bacterially expressed recombinant MBP-PTEN or MBP PTEN ΔTKV were incubated with either GST or GST-SNX27 proteins expressed in bacteria. The association of SNX27 with PTEN was detected by immunoblotting with GST antibody. Expression of all recombinant proteins was shown by coomassie staining. **(F)** Glutathione sepharose beads immobilized with bacterially expressed recombinant GST, GST-SNX27 FL or GST-SNX27 ΔPDZ proteins were incubated with bacterially purified recombinant MBP-PTEN. The association of SNX27 with PTEN was detected by immunoblotting with MBP antibody.

Figure S2

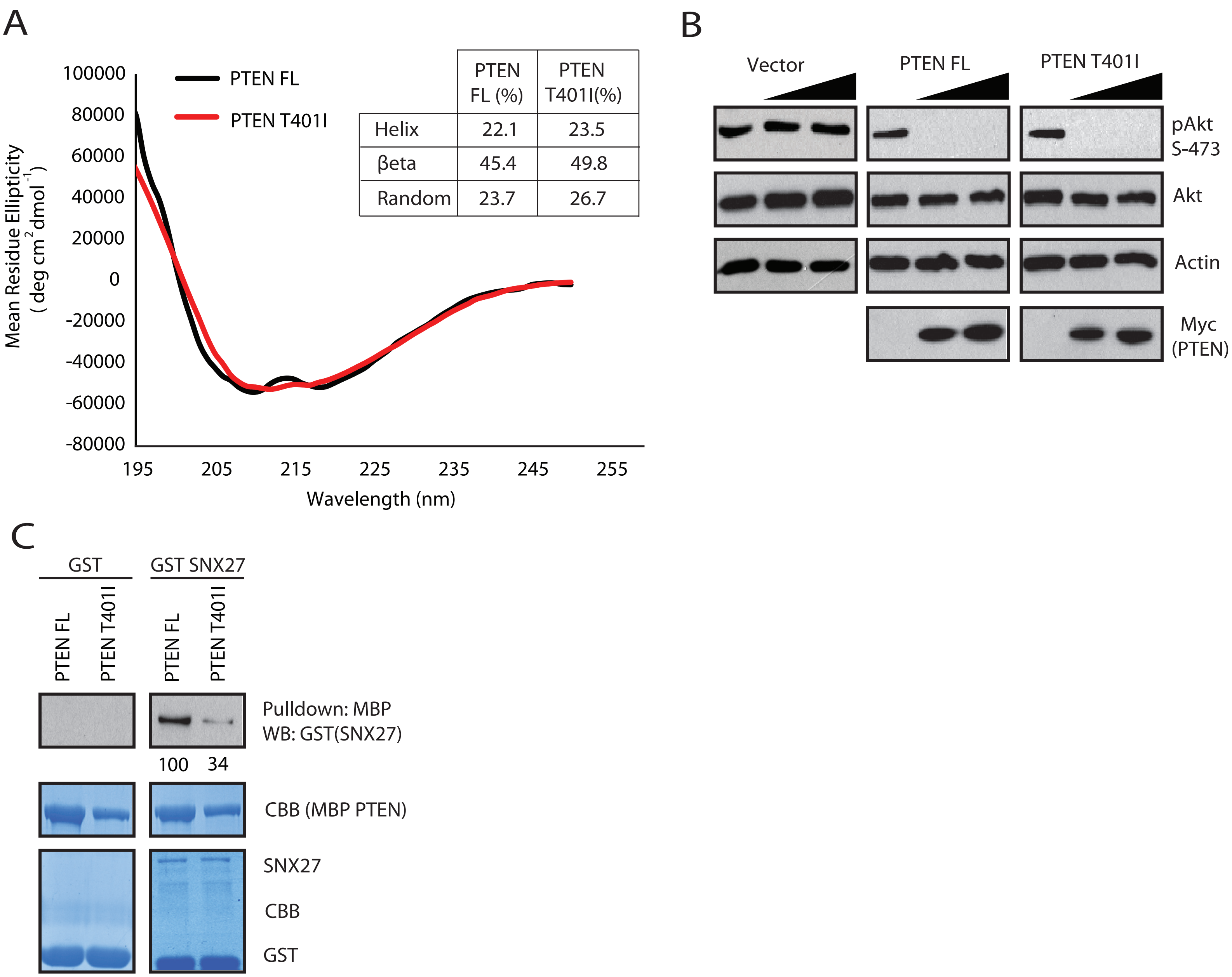

Figure S2, Related to Figure 1.
(A) The CD spectra for full length (FL) PTEN and T401I mutant was shown. **(B)** MDA-MB-231 cells were transfected with vector, PTEN full length (FL) or PTEN T401I mutants. Cell lysates were subjected to immunoblotting analysis and activation of Akt was detected by using a phospho-specific (ser 473) antibody. **(C)** Agarose beads immobilized with bacterially expressed recombinant MBP-PTEN WT or MBP PTEN T401I were incubated with either GST or GST-SNX27 proteins expressed in bacteria. The association of SNX27 with PTEN was detected by immunoblotting with GST antibody. Expression of the all recombinant proteins was shown by coomassie staining.

Figure S3

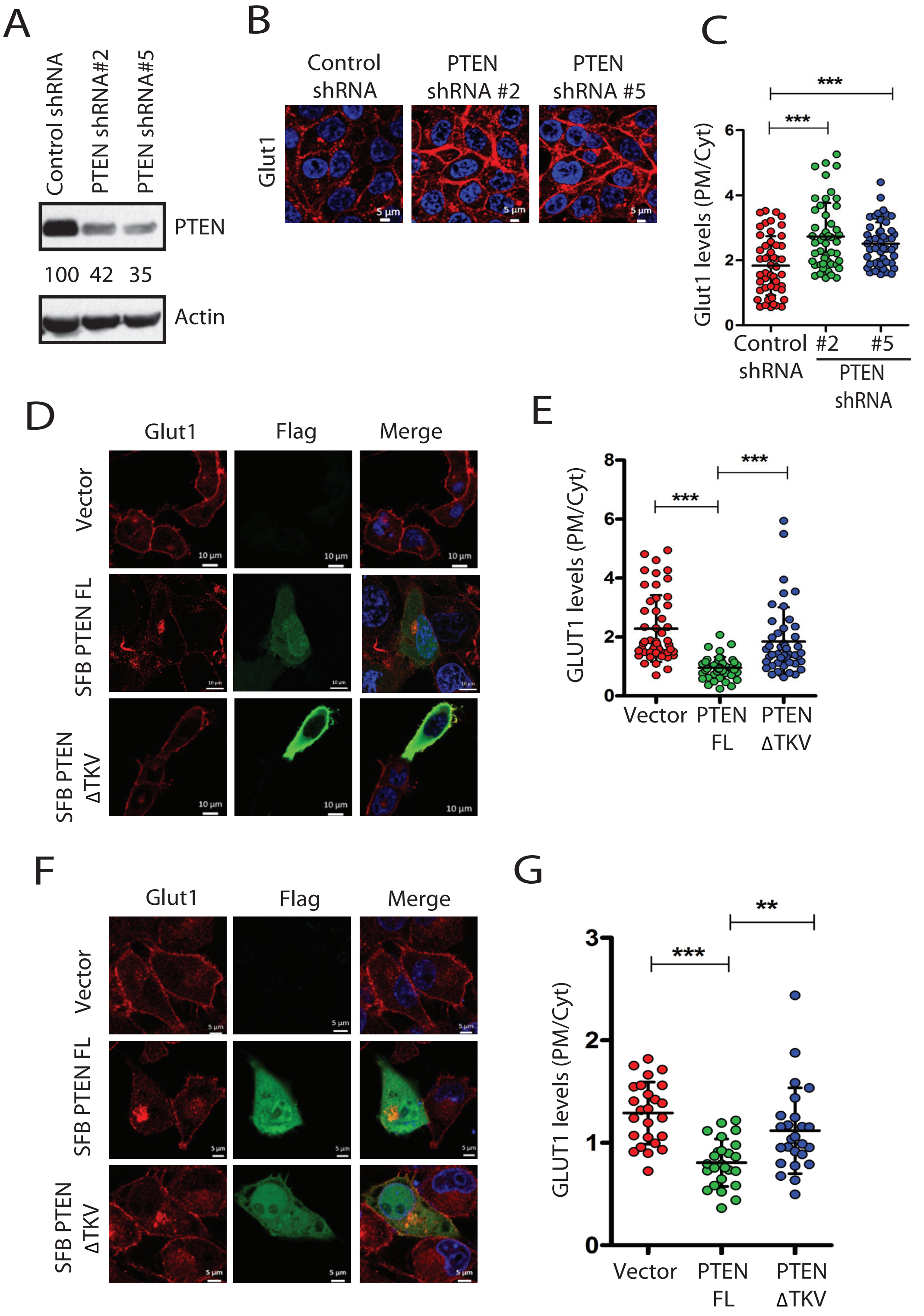

Figure S3, Related to Figure 2.
(A) A representative western blot showing the shRNA mediated knockdown of PTEN in HepG2 cells. **(B)** PTEN depleted HepG2 cells were fixed and imaged using confocal microscope after staining with antibodies against GLUT1. The representative images are shown, Scale bar, 5µm. **(C)** Quantification of plasma membrane (PM) to cytoplasm (Cyt) ratio for GLUT1 intensity per each cell was plotted. Error bars indicate SD (n =50 cells), *** p value (0.001), by Student's t-test. **(D)** Full length (FL) SFB-PTEN or ΔTKV mutant was transfected into MDA-MB231 cells and their effect on GLUT1 membrane levels was determined by confocal imaging after co-staining with Flag and GLUT1 antibodies. The representative images were shown, scale bar, 10 µm. **(E)** Quantification of plasma membrane (PM) to cytoplasm (Cyt) ratio for GLUT1 intensity per each cell was plotted. Error bars indicate SD (n =50 cells), *** p value (0.001), by Student's t-test. **(F)** Full length (FL) SFB PTEN or ΔTKV mutant was transfected into MiaPaCa 2 cells and their effect on GLUT1 membrane levels was determined by confocal imaging after co-staining with Flag and GLUT1 antibodies. The representative images were shown, scale bar, 5 µm. **(G)** Quantification of plasma membrane (PM) to cytoplasm (Cyt) ratio for GLUT1 intensity per each cell was plotted. Error bars indicate SD (n =25 cells), p value (***0.001 or ** 0.01), by Student's t-test.

Figure S4

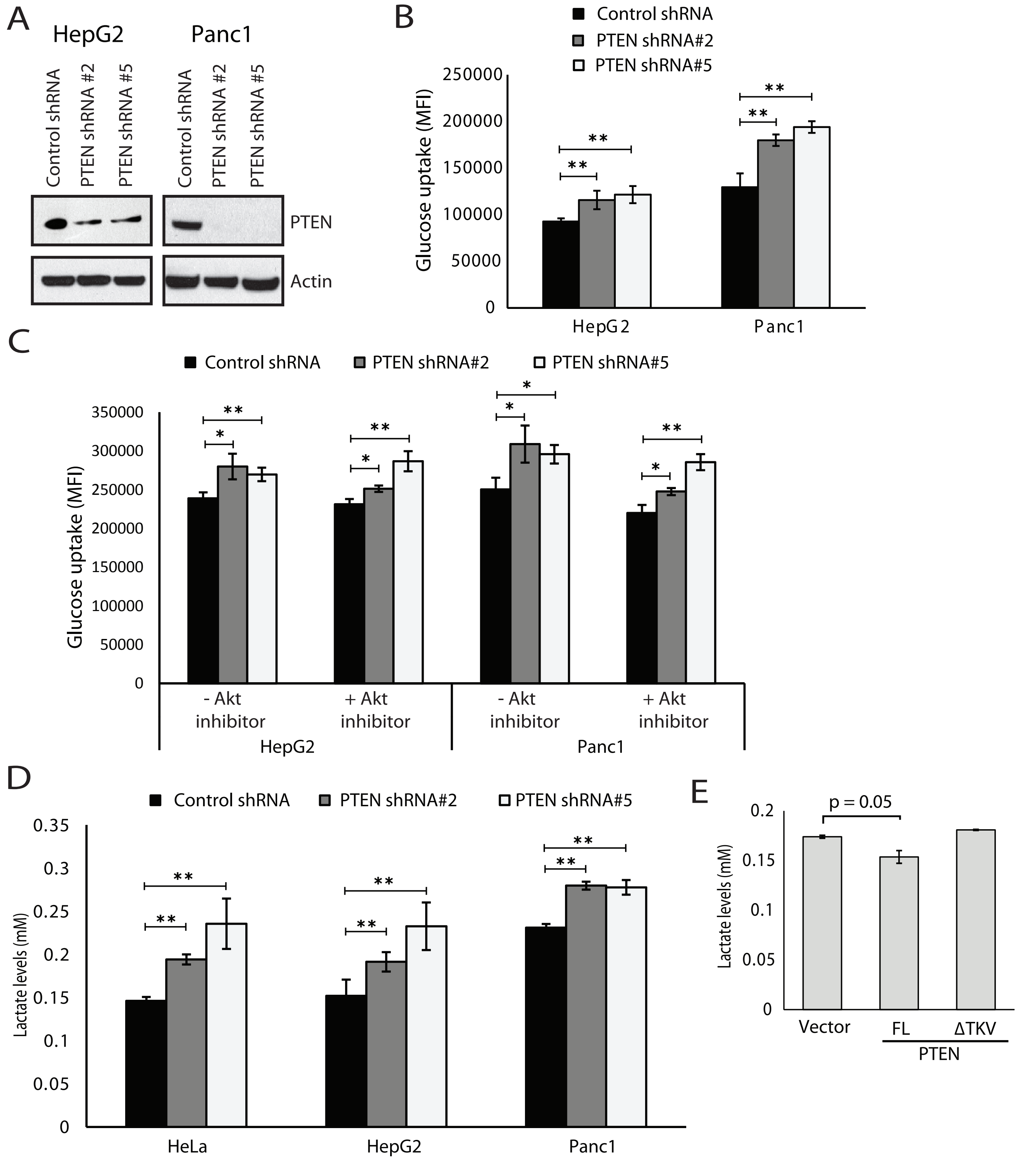

Figure S4, Related to Figure 4.
(A) HepG2 and Panc1 cells transduced with either control or PTEN shRNAs **(B)** were incubated with glucose fluorescent analogue 2-NBDG (100 µg/ml). Glucose uptake in these treated cells was measured. Error bars indicate SD, p value (** 0.01) by Student's t-test. **(C)** HepG2 and Panc1 cells transduced with either control or PTEN shRNAs were treated with Akt inhibitor (MK-2206, 10µM) and glucose uptake in these treated cells was measured. Error bars indicate SD. p value (** 0.01 or * 0.5) by Student's t-test. **(D)** PTEN shRNA transduced cells were lysed and the intracellular lactate concentration was estimated using colorimetry-based assay. Data is expressed as mM concentration of Lactate for three independent experiments. Error bars indicate SD, p value (** 0.01) by Student's t-test. **(E)** MiaPaca2 cells were transfected with SFB- tagged full length (FL) PTEN or PTEN ΔTKV. 24 hours post transfection cells were lysed and intracellular lactate concentration was measured. Data is expressed as mM concentration of Lactate for three independent experiments. Error bars indicate SD, p value (* 0.05) by Student's t-test.

Figure S5

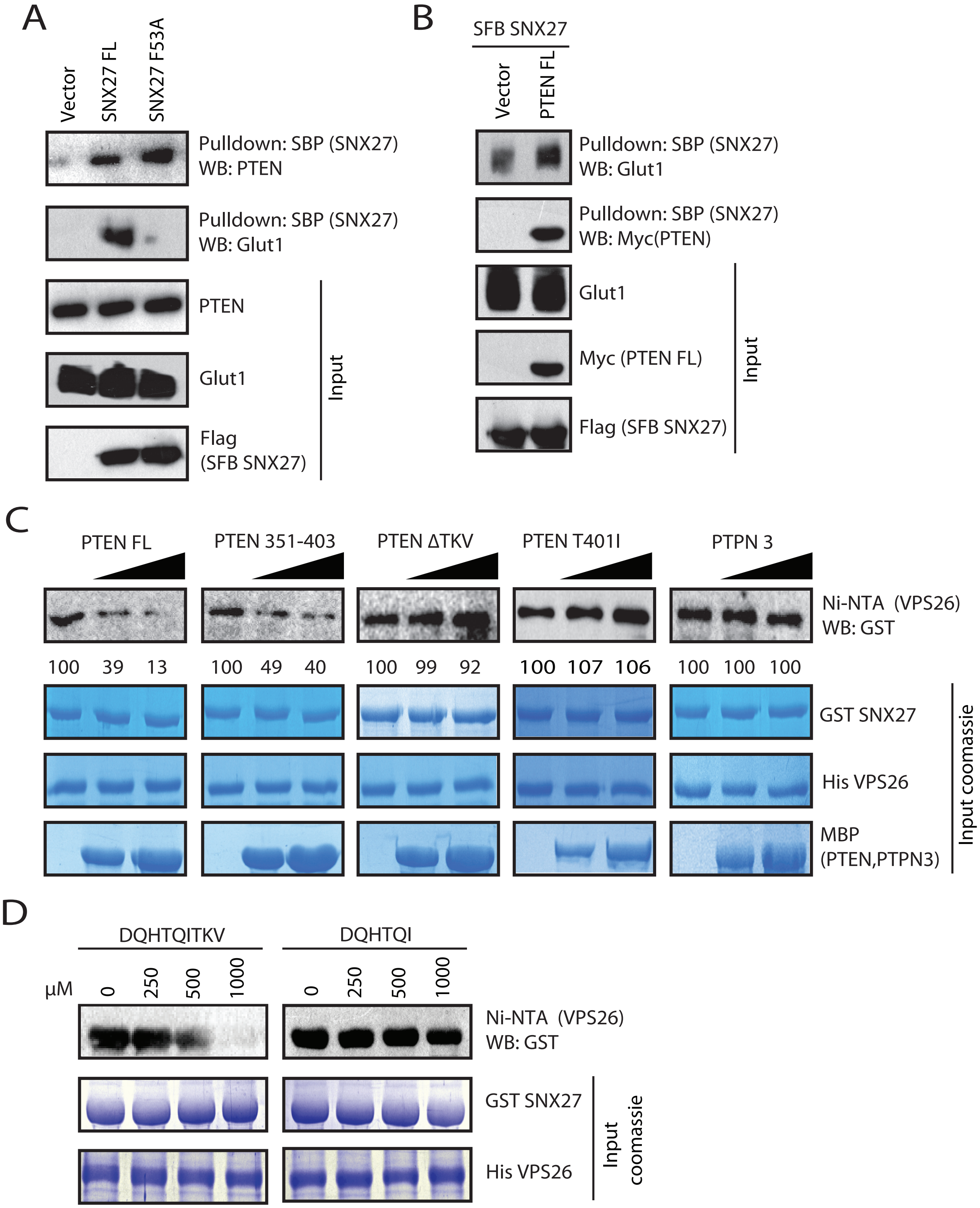

Figure S5, Related to Figure 5.
(A) HEK 293T cells were transfected with the SFB-tagged SNX27 full length (FL) or SNX27 F53A mutant and cell lysates were subjected to pull down with streptavidin beads. The interaction of SNX27 with PTEN and GLUT1 was determined by western blotting with their respective antibodies. **(B)** HEK 293T cells were co-transfected with SFB-tagged SNX27 along with Myc vector or Myc- tagged PTEN and the cell lysates were subjected to pull down with streptavidin beads and interaction of GLUT1 with SNX27 was detected by immunoblotting. **(C)** Bacterially purified recombinant His tagged VPS26 was immobilized on the Ni-NTA beads. Bacterially purified GST-SNX27 was eluted with reduced Glutathione and 5 μM of eluate was incubated with His-tagged VPS26A in presence of increasing PTEN (FL or ΔTKV or T401I or 351-403) or PTPN3. The association of SNX27 with His-VPS26 was determined by western blotting with GST antibody. Expression of all recombinant proteins was shown by coomassie staining. **(D)** The association of recombinant SNX27 with VPS26 in the presence of PTEN derived synthetic peptides was determined by western blotting with GST antibody. Expression of all recombinant proteins was shown by coomassie staining.

Figure S6

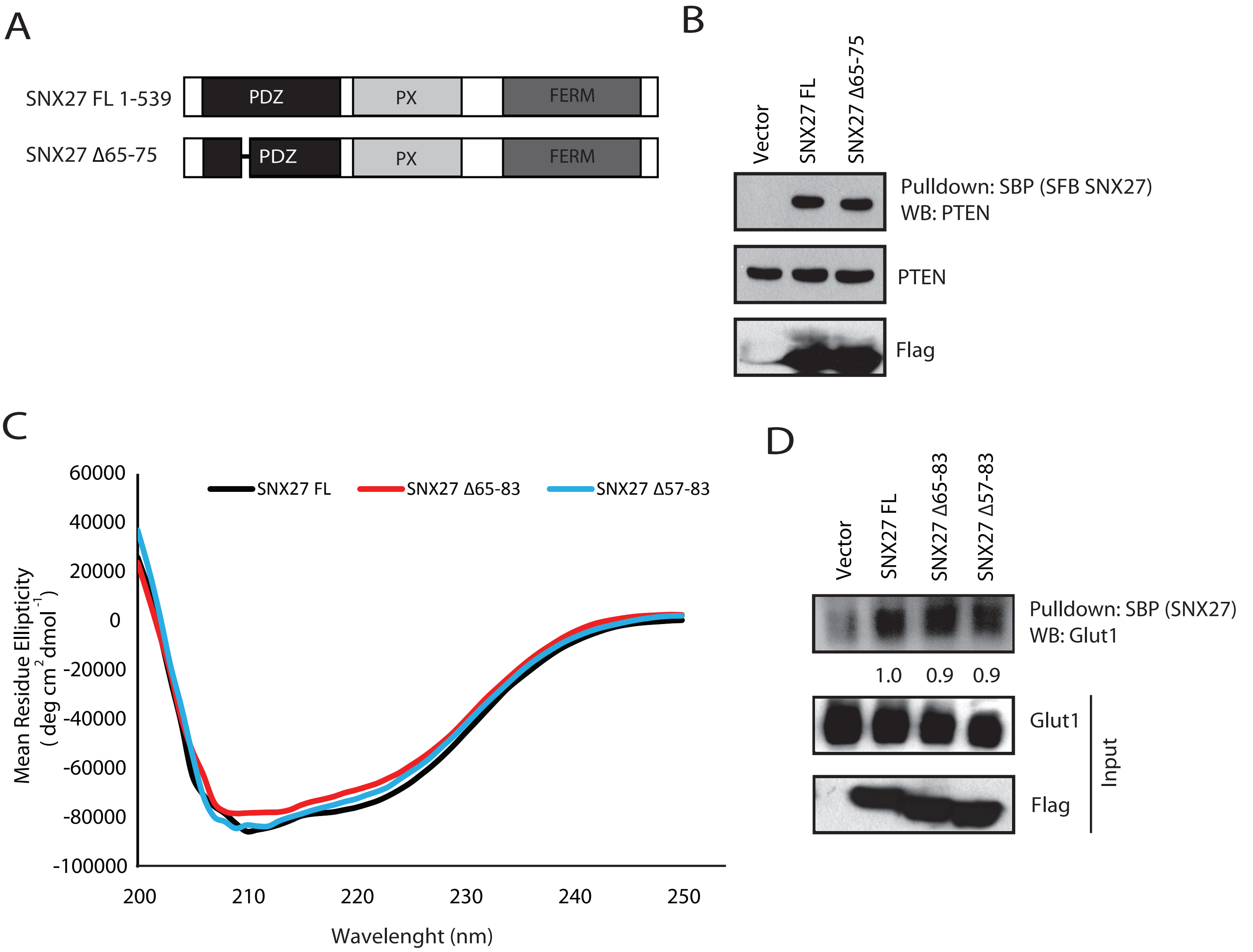

Figure S6, Related to Figure 6.
(A) Schematic representation of N-terminal SFB-tagged SNX27 full length (FL) and SNX27 Δ65-75 mutant. **(B)** Indicated constructs were expressed in HEK 293T cells and the interaction of PTEN with SNX27 was detected by immunoblotting with endogenous PTEN antibody after the cell lysates were pull down with streptavidin beads. The inputs were detected with respective antibodies. **(C)** The CD spectra for full length (FL) SNX27 and the mutants was shown. **(D)** Cells were transfected with the SFB-tagged SNX27 full length (FL) or the indicated mutants and cell lysates were subjected to pull down with streptavidin beads. The interaction of SNX27 with GLUT1 was determined by western blotting.

Figure S7

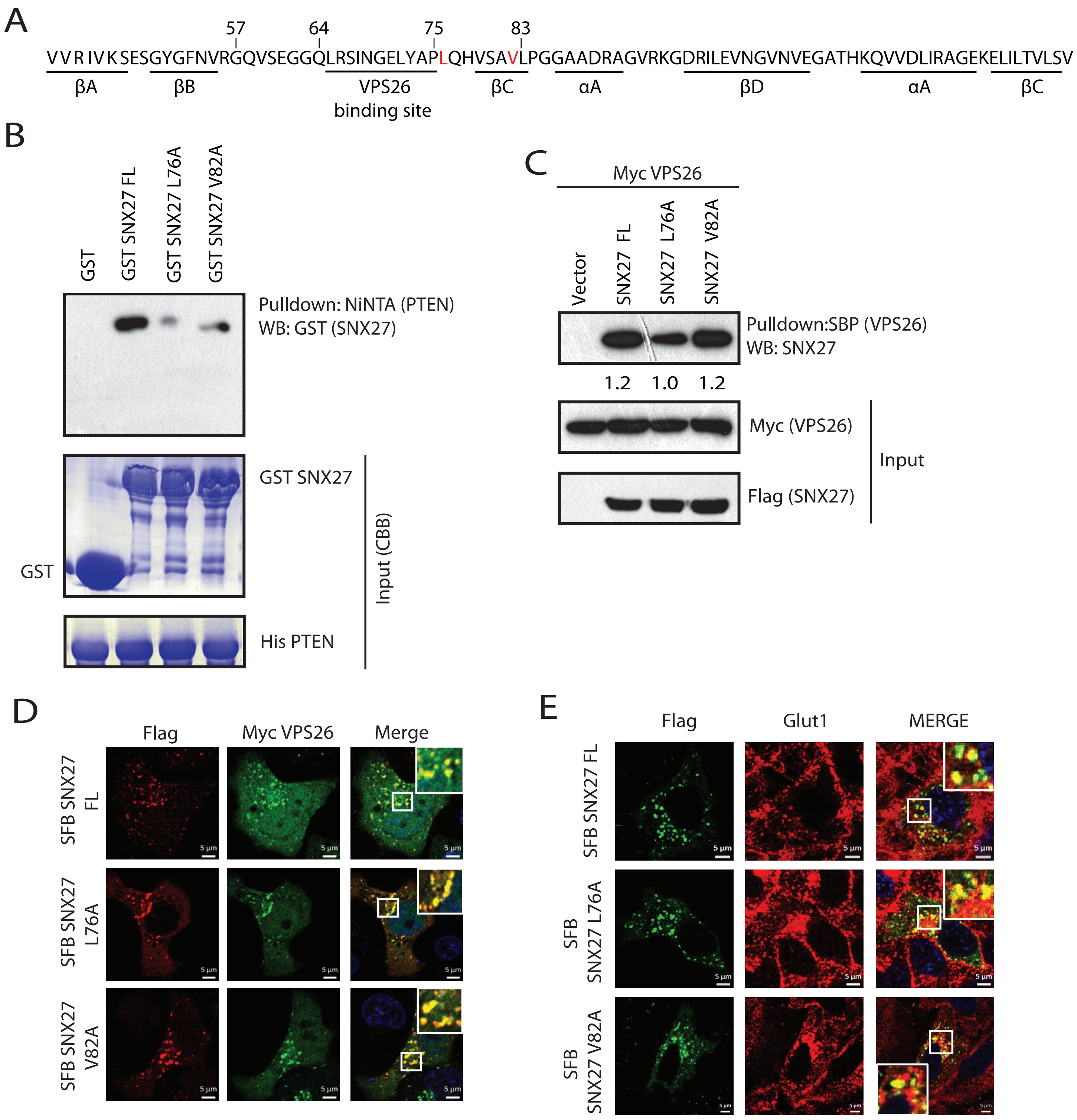

Figure S7, Related to Figure 6.
(A) The sequence of SNX27 PDZ domain is shown along with its VPS26 binding site. The sites of PTEN interaction are highlighted in red (L76 and V82). **(B)** Ni-NTA bound PTEN was incubated with bacterially expressed recombinant GST, GST-SNX27 full length or mutant proteins. The association of SNX27 with PTEN was detected by immunoblotting with GST antibody. Expression of all the recombinant proteins was shown by coomassie staining. **(C)** SFB-tagged SNX27 FL or SNX27 L76A or SNX27 V82A mutants along with Myc VPS26 were transfected in HEK 293T cells. The cell lysates were subjected to pull down with streptavidin beads. The interaction of VPS26 with SNX27 was determined by western blotting Myc antibody. The inputs were blotted with respective antibodies. **(D)** HeLa cells were co-transfected with SFB-SNX27 or mutants along with Myc VPS26. 24 hours post transfection cells were fixed and co-localization of SNX27 and VPS26 was determined by confocal imaging after staining with specific antibodies. **(E)** HeLa cells were co-transfected with SFB-SNX27 or mutants. 24 hours post transfection cells were fixed and co-localization of SNX27 with GLUT1 was determined by confocal imaging after staining with specific antibodies.
